# Dendrobium Officinale‐Derived Carbon Dots Nanozymes Alleviate Colitis by Orchestrating Intestinal Mucus‐Epithelium‐Immune Barriers

**DOI:** 10.1002/advs.202512567

**Published:** 2025-10-17

**Authors:** Chenxi Xu, Xiaoling Huang, Zhichao Deng, Ruiying Wang, Seyedalireza Ghazimirsaeid, Li Yao, Yuanyuan Zhu, Bowen Gao, Junlong Fu, Mingxin Zhang, Mei Yang, Mingzhen Zhang

**Affiliations:** ^1^ Department of Hepatobiliary Surgery the First Affiliated Hospital of Xi’an Jiaotong University Xi'an Shaanxi 710061 China; ^2^ School of Basic Medical Sciences Xi'an Jiaotong University Xi'an Shaanxi 710061 China; ^3^ Department of Gastroenterology People's Hospital of Xinjiang Uygur Autonomous Region Urumqi Xinjiang 830001 China; ^4^ Department of Neurology XD Group Hospital Xi'an Shaanxi 710077 China; ^5^ Department of Gastroenterology The First Affiliated Hospital of Xi'an Medical University Xi'an Shaanxi 710077 China; ^6^ Department of Organ Procurement and Allocation The First Affiliated Hospital of Xi'an Jiaotong University Xi'an Shaanxi 710061 China

**Keywords:** carbon dots, Dendrobium officinale, inflammatory bowel disease, intestinal barrier, nanozymes

## Abstract

Inflammatory bowel disease (IBD) is a chronic inflammatory disorder driven by genetic susceptibility, immune dysregulation, and intestinal barrier dysfunction. Current therapies primarily target immune suppression but show limited efficacy in barrier repair. Here, carbon dots derived from the Chinese herbal medicine *Dendrobium officinale (DO‐CDs)*, which exhibit antioxidant enzyme activity, are synthesized using a hydrothermal method. These DO‐CDs, characterized by an abundance of surface functional groups, are demonstrated to scavenge ROS, suppress M1 macrophage polarization, as well as downregulate pro‐inflammatory cytokine expression. In both acute and chronic colitis models, DO‐CDs demonstrate multimodal barrier‐repair properties. It is shown that DO‐CDs can notably restore colon length, reduce the infiltration of inflammatory cells, and enhance both the quantity of goblet cells and the expression of mucins. Furthermore, the expression of intestinal epithelial tight junction proteins is significantly upregulated following DO‐CDs treatment, thereby effectively strengthening the intestinal epithelial barrier function. Importantly, DO‐CDs modulate the Th1/Treg ratio by downregulating the proportions of dendritic cells and M1 macrophages, thus reestablishing intestinal immune homeostasis. These coordinated actions on the mucus‐epithelium‐immune triad demonstrate the unique capacity of DO‐CDs for holistic barrier reconstruction. The work provides a mechanistic foundation for herbal precursor‐derived carbon dots as multi‐target therapeutics in IBD.

## Graphical abstract

Dendrobium officinale‐derived carbon dots (DO‐CDs) exhibiting enzyme‐mimetic activities are synthesized via a hydrothermal approach. In the colitis model, DO‐CDs effectively facilitated the repair of mucosal, epithelial, and immune barriers by upregulating the expression of mucins and tight junction proteins, as well as re‐establishing intestinal immune homeostasis. These actions contributed to the mitigation of colitis progression, demonstrating significant therapeutic efficacy.

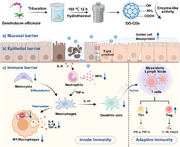

## 1 Introduction

Inflammatory bowel disease (IBD), primarily comprising Crohn's disease (CD) and ulcerative colitis (UC),^[^ ^1^ ^]^ represents a chronic inflammatory disorder impacting the gastrointestinal tract. Characterized by a prolonged course and frequent relapses,^[^ ^2^ ^]^ it may eventually lead to weakened gastrointestinal function and even life‐threatening complications.^[^ ^3^ ^]^ The pathogenesis of IBD is related to complex interactions among genetic, environmental, microbial, and immune factors,^[^ ^4^ ^]^ representing the result of dysregulated immune responses in genetically susceptible individuals to changes in the intestinal environment.^[^ ^5^ ^]^ Studies on genes and susceptibility gene loci of IBD have shown that multiple pathways, such as intestinal barrier function, innate and adaptive immunity regulation, microbial factors, and oxygen‐free radical production, play important roles in maintaining intestinal homeostasis.^[^ ^6^ ^]^ The disruption of redox balance in the intestine, along with the subsequent impairment of the intestinal barrier, is crucial for the pathogenesis of IBD.^[^ ^7^ ^]^

The intestinal barrier function is composed of the mucosal barrier, epithelial barrier, immune barrier, and biological barrier, which collectively regulate intestinal physiological functions and protect the host against pathogenic invasions.^[^ ^8^ ^]^ Current therapeutic strategies for IBD primarily focus on suppressing hyperactivated immune responses through conventional pharmacological agents. For instance, 5‐aminosalicylic acid (5‐ASA) alleviates inflammation by inhibiting nuclear factor κB (NF‐κB) signaling pathways,^[^ ^9^ ^]^ while monoclonal antibody therapies (e.g., anti‐TNF‐α and anti‐IL‐12/23 antibodies) exert therapeutic effects by neutralizing pro‐inflammatory cytokines.^[^ ^10^ ^]^ Nonetheless, the restoration of barriers necessitates multifactorial mechanisms, encompassing stem cell renewal, mucus‐microbiota interactions, and the reconstruction of tight junctions.^[^ ^11^ ^]^ These complex processes are insufficiently addressed by single‐target anti‐inflammatory agents.

To address these limitations, nanomaterials have emerged as innovative strategies for multidimensional intestinal barrier repair in IBD treatment.^[^ ^12^ ^]^ For example, tungsten‐encapsulated zinc nanoparticles with bilayer structures enhance epithelial barrier integrity via zinc ion‐mediated metallothionein expression while modulating gut microbiota through tungsten elements, achieving synergistic regulation of intestinal homeostasis.^[^ ^13^ ^]^ Similarly, chondroitin sulfate‐modified high‐specificity and persistent targeting Ta_2_C nanomedicine restores oxidative stress‐immunoinflammatory‐barrier equilibrium in local microenvironments by scavenging reactive oxygen species (ROS).^[^ ^14^ ^]^ Despite these promising advancements, the clinical translation of nanomaterials remains hindered by an insufficient understanding of their potential biotoxicity and underlying therapeutic mechanisms.^[^ ^15^ ^]^ Therefore, exploration and development of safe and effective nanomaterial‐based strategies are crucial for advancing IBD therapeutics.

Carbon dots (CDs), a novel class of 0D photoluminescent nanomaterials characterized by sub‐10 nm particle dimensions, were first isolated through the purification of single‐walled carbon nanotubes in 2004.^[^ ^16^ ^]^ Their small size and surface‐abundant functional groups endow carbon dots with numerous advantages,^[^ ^17^ ^]^ including tunable fluorescence emission/excitation wavelengths, excellent photostability, water solubility, biocompatibility, and enzyme‐like activities.^[^ ^18^ ^]^ These properties have garnered significant attention in biomedical research.^[^ ^19^ ^]^ Conventional chemically‐based synthesis of CDs often involves the release of toxic byproducts such as oxidative stress, ROS, and metal ions.^[^ ^20^ ^]^ In alignment with the principles of green chemistry, biomass‐derived precursors have emerged as promising alternatives due to their low toxicity, abundant heteroatoms, and excellent biocompatibility.^[^ ^21^ ^]^ To date, numerous CDs synthesized from eco‐friendly precursors have been reported for applications including anti‐inflammatory treatment and tissue repair.^[^ ^22^ ^]^ For instance, Wei et al. synthesized fluorescent CDs using the natural Chinese herb *Gynostemma pentaphyllum* as a precursor, demonstrating their ability to enhance the mRNA expression of antioxidant genes and alleviate hydrogen peroxide‐induced oxidative damage.^[^ ^23^ ^]^ Similarly, Deng et al. developed Hy‐CDs from *honeysuckle* via a hydrothermal method, which effectively treated acute lung injury by suppressing Caspase11/GSDMD‐dependent pyroptosis.^[^ ^24^ ^]^ Traditional Chinese herbs contain abundant, diverse sugars, acids, phenolic compounds, and glycosides. These molecules can dehydrate at high temperatures to generate furfural derivatives, which subsequently polymerize into aromatic structures and carbonate into carbon cores,^[^ ^25^ ^]^ making them ideal candidates for synthesizing CDs. However, to advance the clinical application of traditional Chinese medicine carbon dots, it is still necessary to address issues such as batch differences, unclear structure‐activity relationships, and difficulty in identifying active ingredients. In addition, rigorous testing is required for long‐term in vivo safety assessment, including metabolic pathways and potential accumulation in organs.

**Figure 1:**
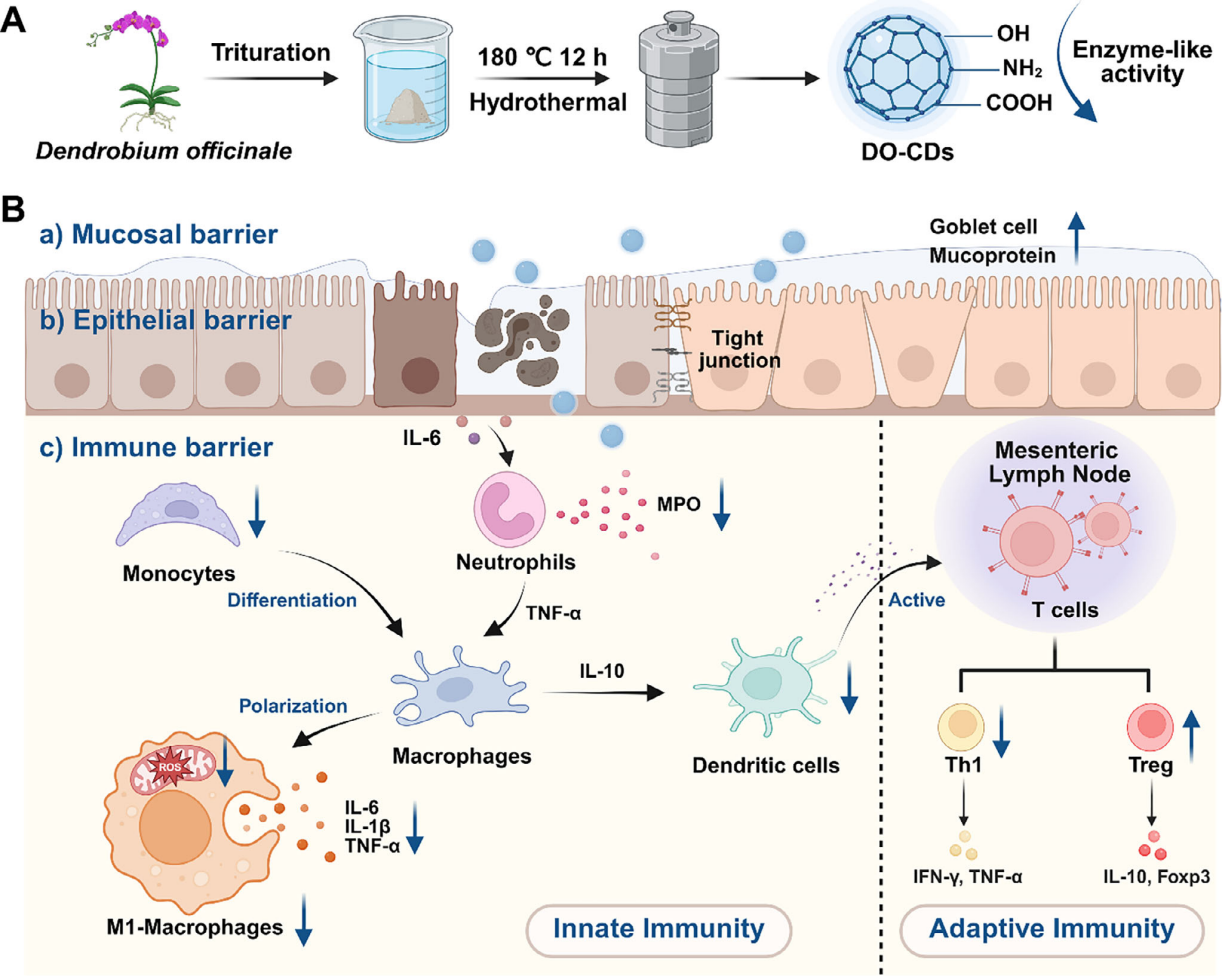
The diagram depicts the process of synthesizing DO‐CDs and their biological mechanism for treating colitis. A) DO‐CDs are synthesized through a hydrothermal approach, featuring surface‐functionalized hydroxyl, amino, and carbonyl groups. These chemical groups confer enzyme‐like activities to the DO‐CDs. B) DO‐CDs alleviate the progression of colitis by restoring multiple critical barriers: a) Mucosal barrier (Increasing goblet cell numbers and enhancing mucin expression); b) Epithelial barrier (Repairing the expression of intercellular tight junction proteins); c) Immune barrier (Modulation of both innate and adaptive immunity).

In this study, Dendrobium Officinale (DO), a traditional medicinal herb recognized for its properties of enhancing gastrointestinal mucosal thickness and stimulating gastric fluid production, was chosen as the precursor for the synthesis of carbon dots (DO‐CDs). The resulting DO‐CDs exhibited potent antioxidant enzyme‐like activities (**Figure** **1**A). We found DO‐CDs significantly downregulated the transcriptional expression of pro‐inflammatory cytokines by targeting the TNF‐mediated NF‐κB signaling pathway through in vitro and in vivo experiments. Additionally, they effectively inhibited the M1‐type macrophage polarization, ultimately alleviating inflammatory responses. In DSS‐induced colitis models, DO‐CDs modulated the dynamic equilibrium between the innate and adaptive immune systems, thereby maintaining intestinal immune microenvironment homeostasis. By reducing inflammatory cell infiltration in intestinal tissues, promoting colonic goblet cell proliferation, and upregulating tight junction protein expression, DO‐CDs markedly suppressed colitis progression through the restoration of the mucosal barrier, epithelial barrier, and immune barrier in the colon. Collectively, DO‐CDs demonstrated multi‐target therapeutic efficacy in both acute and chronic colitis. This study provides a theoretical foundation and practical reference for the preparation of CDs from herbal precursors and their application in treating inflammatory diseases (Figure 1B).

## 2 Results and Discussion

### 2.1 Synthesis and Characterization of DO‐CDs

**Figure 2:**
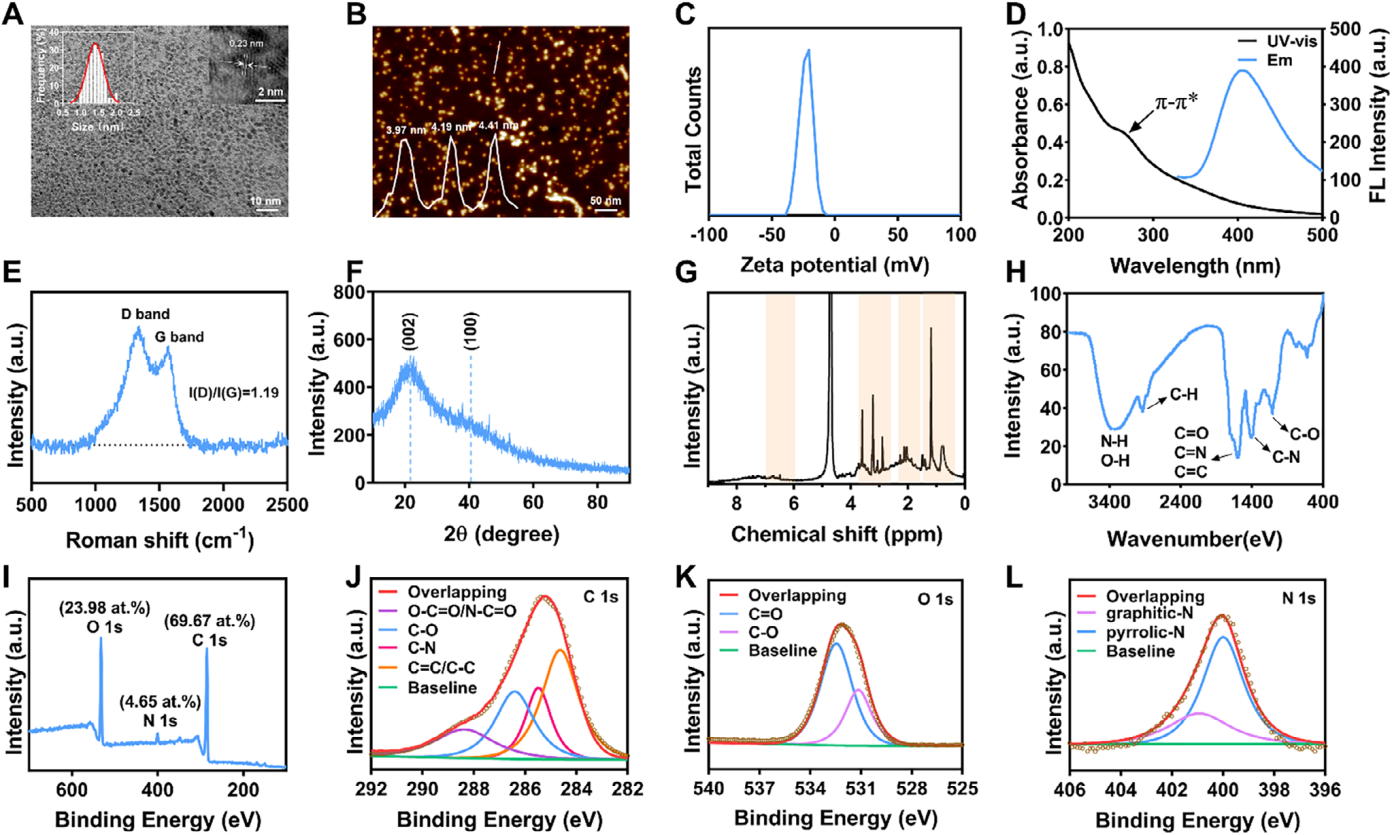
Characterization of DO‐CDs. A) Images of TEM (scale bar: 10 nm) and HR‐TEM (scale bar: 2 nm), and size distribution histogram of DO‐CDs. B) AFM and distribution height of DO‐CDs (scale bar: 50 nm). C) Zeta potential of DO‐CDs. D) UV (black) and fluorescence (blue) spectra of DO‐CDs. E) Raman spectrum of DO‐CDs. F) XRD pattern of DO‐CDs. G) ^1^H‐NMR spectrum of DO‐CDs. H) FT‐IR spectrum of DO‐CDs. I) XPS survey spectrum of DO‐CDs. High‐resolution XPS spectra of C 1s J), O 1s K), and N 1s L) for DO‐CDs.

Using the Chinese herbal medicine *Dendrobium officinale* as the precursor of carbon materials, DO‐CDs were synthesized through a simple hydrothermal method. We first characterized the macroscopic morphology of DO‐CDs. Transmission electron microscopy (TEM) results revealed that DO‐CDs exhibited good dispersion and quasi‐spherical morphology, with an average diameter of 1.3 ± 0.3 nm based on statistical analysis of particle size distribution. High‐resolution TEM (HRTEM) showed lattice fringes in DO‐CDs with a spacing of 0.23 nm, aligned with the (100) plane of graphite, suggesting a graphite‐like crystalline structure (**Figure** **2**A).^[^ ^26^ ^]^ Atomic force microscopy (AFM) results confirmed the monodispersity of DO‐CDs, with heights consistent with their average particle size, indicating spherical or quasi‐spherical morphology (Figure 2B). To evaluate the stability of DO‐CDs, the zeta potential was measured continuously for 7 days. The results show that there are no significant changes in zeta potential (Figure 2C; Figure S1A, Supporting Information). The small particle size of DO‐CDs facilitates their penetration through biological barriers.^[^ ^27^ ^]^ Optical characterization showed that the UV–vis absorption spectrum of DO‐CDs exhibited a band ≈280 nm, attributed to π–π* transitions from sp^2^‐hybridized structures.^[^ ^28^ ^]^ Under optimal excitation at 350 nm, the fluorescence spectrum displayed an emission peak centered at 438 nm (Figure 2D; Figure S1, Supporting Information).^[^ ^29^ ^]^ The above results demonstrate the successful synthesis of DO‐CDs.^[^ ^30^ ^]^

CDs are typically described as small stacked graphene layers that self‐assemble into quasi‐spherical structures, comprising two main components: a carbon atomic core with sp^2^/sp^3^ amorphous hybridization and a peripheral layer abundant in oxygen‐, nitrogen‐, and sulfur‐bearing moieties coupled with macromolecular chain assemblies.^[^ ^31^ ^]^ Therefore, we next explored the surface structure of DO‐CDs. Raman spectroscopy revealed two characteristic peaks at 1335.11 cm^−1^ (D‐band, disordered defects) and 1565.07 cm^−1^ (G‐band, graphitic structure), with an I(D)/I(G) ratio of 1.19, indicating a defect‐rich graphitic surface, which is associated with the presence of hybrid groups on their surface (Figure 2E).^[^ ^32^ ^]^ X‐ray diffraction (XRD) analysis revealed a broad diffraction peak at 22°, which corresponds to the (002) plane of graphitic carbon (Figure 2F). This observation suggests that the carbon structure of DO‐CDs predominantly consists of defect‐rich nano‐graphitic crystallites, aligning with the notion of an incomplete graphitization process occurring during the hydrothermal carbonization of biomass. Nuclear magnetic resonance hydrogen spectroscopy (^1^H NMR spectra) displayed peaks between 6.00 ppm and 7.00 ppm (olefinic protons), 3.00–4.00 ppm (hydroxyl‐attached carbons), 1.0–1.8 ppm (aliphatic hydrocarbons), and 2.0–2.5 ppm (α‐H of carboxyl/amino groups) (Figure 2G).^[^ ^33^ ^]^ Fourier transform infrared spectroscopy (FT‐IR) analysis identified key functional groups: a broad peak at 3350.8 cm^−1^ (N‐H stretching), 2930.4 cm^−1^ (C─H bending), 1616.1 cm^−1^ (C = O), 1600 cm^−1^ (C = N), and 1120 cm^−1^ (C─O) (Figure 2H).^[^ ^34^ ^]^ X‐ray photoelectron spectroscopy (XPS) confirmed the presence of carbon, nitrogen, and oxygen (Figure 2I). High‐resolution C1s spectra revealed four components at 288.8 eV (O─C = O/N‐C = O), 286.5 eV (C─O), 285.7 eV (C─N), and 284.6 eV (C = C/C─C) (Figure 2J). O1s spectra showed peaks at 533 eV (C─O) and 531 eV (C = O) (Figure 2K), while N1s spectra indicated graphitic nitrogen (400.9 eV) and pyrrolic nitrogen (399.9 eV) (Figure 2L). Collectively, these structural analyses demonstrate that DO‐CDs possess a defect‐rich graphitic framework with abundant surface functional groups (amino, carboxyl, carbonyl, etc.), which may confer potential pharmacological activities and catalytic properties.^[^ ^35^ ^]^

### 2.2 Antioxidant Capacity of DO‐CDs

**Figure 3:**
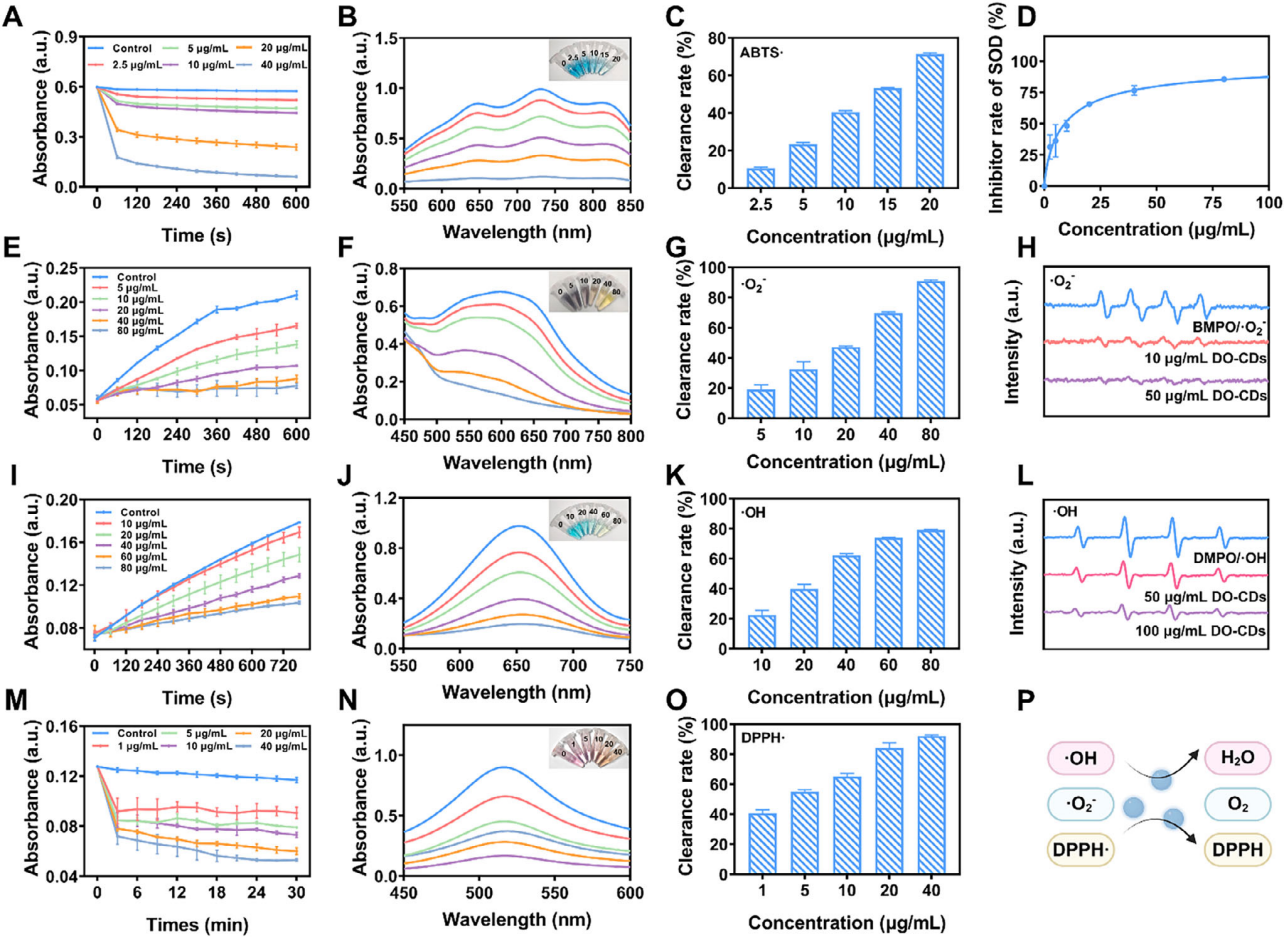
Antioxidant capacity of DO‐CDs. A) Kinetics profiles, B) UV–vis absorption spectra, and C) 10‐min elimination efficiency of ⋅ABTS^+^ radicals under varying DO‐CDs concentrations. D) SOD‐like activities of DO‐CDs. E) Kinetics profiles, F) UV–vis absorption spectra, G) 10‐min scavenging ratios, and H) ESR signals for ⋅O_2−_ radical scavenging. I) Kinetics profiles, J) UV–vis spectral patterns, K) 12‐min clearance percentages, and L) ESR signals for ⋅OH radical scavenging. M) Kinetics profiles, N) UV–vis absorption spectra, O) 30‐min inhibition rates of DPPH·. P) Antioxidant mechanism schematic. (*n* = 3).

The properties of CDs arise from the complex interplay of their core structure, defects, and surface states. Beyond their diverse fluorescence emissions, another notable characteristic of CDs, their antioxidant capacity, has garnered significant attention.^[^ ^36^ ^]^ We first evaluated the total antioxidant capacity of DO‐CDs using the 2,2'‐Azinobis‐(3‐ethylbenzthiazoline‐6‐sulphonate) ABTS assay. The ABTS· radical was prepared by reacting ABTS with an oxidizing agent overnight. Different concentrations of DO‐CDs were then co‐incubated with ABTS·. Next, the absorbance of the solution was measured at 405 nm every 60 s. The results showed that with the increase of DO‐CDs concentration, the blue color of the mixture gradually faded, accompanied by a reduction in absorbance, indicating excellent overall antioxidant activity of DO‐CDs (**Figure** **3**A–C).

The ROS‐scavenging capacity of DO‐CDs was further examined by evaluating their scavenging efficacy toward·O_2−_, ·OH, and DPPH· radicals. The ·O_2−_ scavenging capacity was measured via the nitroblue tetrazolium (NBT) method and commercial superoxide dismutase (SOD) assay kit (WST‐1). Riboflavin‐generated ·O_2−_ reduces NBT to dark purple formazan under aerobic conditions. Kinetic assays with varying concentrations of DO‐CDs revealed that the absorbance at 560 nm increased over time in the absence of DO‐CDs, reflecting substantial ·O_2−_ generation. DO‐CDs notably inhibited ·O_2−_ generation in a concentration‐dependent fashion, with a SOD‐like activity value of 461.37 U mg^−1^. (Figure 3D–G). Using the 3,3',5,5'‐Tetramethylbenzidine (TMB) method to evaluate the ·OH scavenging ability. The TMB substrate turns blue under ·OH influence, and the addition of DO‐CDs lightened the solution color, with reduced absorbance at 652 nm. When the concentration of DO‐CDs reaches 80 µg mL^−1^, the scavenging rate of ·OH is ≈80 % (Figure 3I–K). For nitrogen‐containing radicals, the DPPH· assay demonstrated that DO‐CDs effectively scavenged DPPH·. The purple DPPH· ethanolic solution, with maximum absorption at 517 nm, faded upon interaction with DO‐CDs, as evidenced by kinetic and UV–vis spectral analyses. Higher concentrations of DO‐CDs correlated with greater color fading. Statistical results show that the scavenging rate of DPPH· by DO‐CDs at a concentration of 40 µg mL^−1^ can approach 100 % (Figure 3M–O). Finally, ESR spectroscopy further verified the concentration‐dependent scavenging effects of DO‐CDs on both ·O_2−_ and ·OH radicals (Figure 3H,L). These findings collectively demonstrate that DO‐CDs possess robust antioxidant capabilities, suggesting their therapeutic potential in colitis diseases associated with excessive ROS production (Figure 3P).

### 2.3 Cellular Antioxidative and Anti‐Inflammatory Properties of DO‐CDs

**Figure 4:**
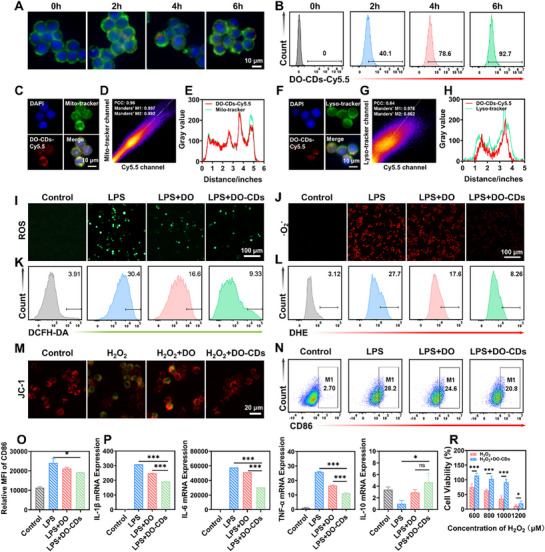
Evaluation of antioxidant and anti‐inflammatory effects of DO‐CDs at the cellular level. A) Cellular uptake of DO‐CDs in RAW264.7 cells (scale bar: 10 µm). B) Flow cytometry detection of DO‐CDs‐cy5.5 in RAW264.7 cells. Fluorescence micrographs demonstrating DO‐CD co‐localization with mitochondria C) and lysosomes F) (Scale bar: 10 µm). Quantitative analysis of organelle co‐distribution: Pearson's correlation coefficient and Mander's overlap coefficient for mitochondrial D) and lysosomal G) colocalization. Plot profile of DO‐CDs within mitochondria E) and lysosomal compartments H). Intracellular ROS visualization in RAW264.7 macrophages: DCFH‐DA I) and DHE J) fluorescent probes (Scale bar: 100 µm). Flow cytometric quantification of intracellular ROS levels using DCFH‐DA K) and superoxide anion detection via DHE L). M) JC‐1‐stained RAW264.7 cell fluorescence images (scale bar: 20 µm). N) Flow cytometric analysis of M1‐phenotype macrophages and CD86 labeling quantification O). P) qPCR results for inflammatory factors IL‐1β, IL‐6, TNF‐α, and IL‐10 under different treatments in Raw264.7 cells. R) Cell viability following H_2_O_2_ treatment. (*n* = 3). Statistical significance was indicated as ^*^ *p* < 0.05, ^**^ *p* < 0.01, ^***^ *p* < 0.001.

Cellular anti‐oxidative and anti‐inflammatory efficacy was systematically benchmarked against DO extract, building on the ROS/RNS‐scavenging capacity of DO‐CDs. Cellular uptake is the first step for DO‐CDs to exert their effects. Since the intrinsic fluorescence of DO‐CDs overlaps with the DAPI fluorescence channel, we labeled DO‐CDs with the red fluorescent probe Sulfo‐Cyanine5.5 (Cy5.5) to track cellular internalization. Uptake of DO‐CDs‐Cy5.5 by RAW264.7 cells was monitored over time, showing a gradual increase in internalization, reaching 92.7 % at 6 h (**Figure** **4**A,B). Subcellular localization of DO‐CDs was then examined. Mito‐Tracker and Lyso‐Tracker were used to label mitochondria and lysosomes. The Pearson correlation coefficient for DO‐CDs co‐localization with mitochondria was 0.96 (Figure 4C–E), while co‐localization with lysosomes yielded a coefficient of 0.64 (Figure 4F–H). These results indicate that DO‐CDs possess both active mitochondrial targeting ability and lysosomal escape characteristics. As mitochondria serve as the core sites of oxidative stress, this property enables DO‐CDs to efficiently scavenge ROS at their production sites.^[^ ^37^ ^]^ Meanwhile, the potential of DO‐CDs to evade enzymatic degradation through lysosomal escape prolongs their effective duration within cells.

To evaluate the antioxidant effects of DO‐CDs in RAW264.7 cells, we employed DCFH‐DA and DHE probes to assess intracellular ROS scavenging. DCFH‐DA interacts with ROS to produce fluorescent 2′,7′‐dichlorofluorescein (DCF), while DHE specifically detects superoxide anions. In DCFH‐DA assays, H_2_O_2_‐treated cells exhibited strong green fluorescence, confirming elevated ROS levels. Co‐incubation with DO‐CDs significantly reduced this fluorescence, demonstrating effective ROS clearance (Figure 4I). Flow cytometry further quantified this effect, revealing that DO‐CDs outperformed DO extract in ROS scavenging (Figure 4K). Similar results were observed in DHE assays (Figure 4J,L). The above results indicate that DO‐CDs have great antioxidant capacity at the cellular level. Given that DO‐CDs can co‐localize with mitochondria and demonstrate the ability to eliminate ROS within cells, we employed JC‐1 as the fluorescent probe to detect the mitochondrial membrane potential (MMP) changes. At high MMP, JC‐1 aggregates in the mitochondrial matrix, emitting red fluorescence. In contrast, when MMP reduces or collapses, JC‐1 remains as monomers and emits green fluorescence. Using an upright microscope, we observed that H_2_O_2_‐treated cells exhibited weaker red fluorescence and stronger green fluorescence, indicating MMP collapse. After co‐incubation with DO‐CDs, the red fluorescence intensity increased, suggesting partial restoration of MMP (Figure 4M). DO‐CDs are capable of repairing mitochondrial damage and maintaining normal mitochondrial function, which provides key mechanistic evidence for their application in oxidative stress‐related diseases.

Beyond ROS generation, macrophage polarization plays a critical role in inflammatory diseases. LPS‐induced M1‐type macrophage polarization was assessed via CD86 fluorescence labeling. DO‐CDs treatment reduced the proportion of M1‐type macrophages, partially suppressing LPS‐driven polarization (Figure 4N,O). Compared with M1‐type macrophages, M2‐type macrophages play a crucial role in maintaining the body's immune homeostasis and promoting tissue repair. In this study, by detecting CD206, a specific marker on the surface of M2‐type macrophages, we analyze whether DO‐CDs can promote the transformation of macrophages into M2‐type cells to alleviate the inflammatory response. The results of flow cytometry showed that after intervention with DO‐CDs, the number of M2‐type macrophages increased significantly compared with the LPS group (Figure S2, Supporting Information). Additionally, qPCR analysis revealed that DO‐CDs significantly downregulated LPS‐induced pro‐inflammatory cytokines, such as TNF‐α, IL‐6, and IL‐1β at the RNA level (Figure 4P). Comparative analysis revealed DO‐CDs' superior *ex vivo* antioxidative and anti‐inflammatory capacities relative to DO extracts. Subsequent evaluation confirmed their cytoprotective efficacy against oxidative damage in model systems. H_2_O_2_‐induced cell death was mitigated by DO‐CDs co‐incubation, as evidenced by increased cell viability, confirming their ability to counteract oxidative damage (Figure 4R). In summary, DO‐CDs exert protective effects against mitochondrial damage and cell death induced by oxidative stress, offering mechanistic proof for the utilization of DO‐CDs in the precise treatment of colitis.

### 2.4 The Biological Mechanisms of DO‐CDs

**Figure 5:**
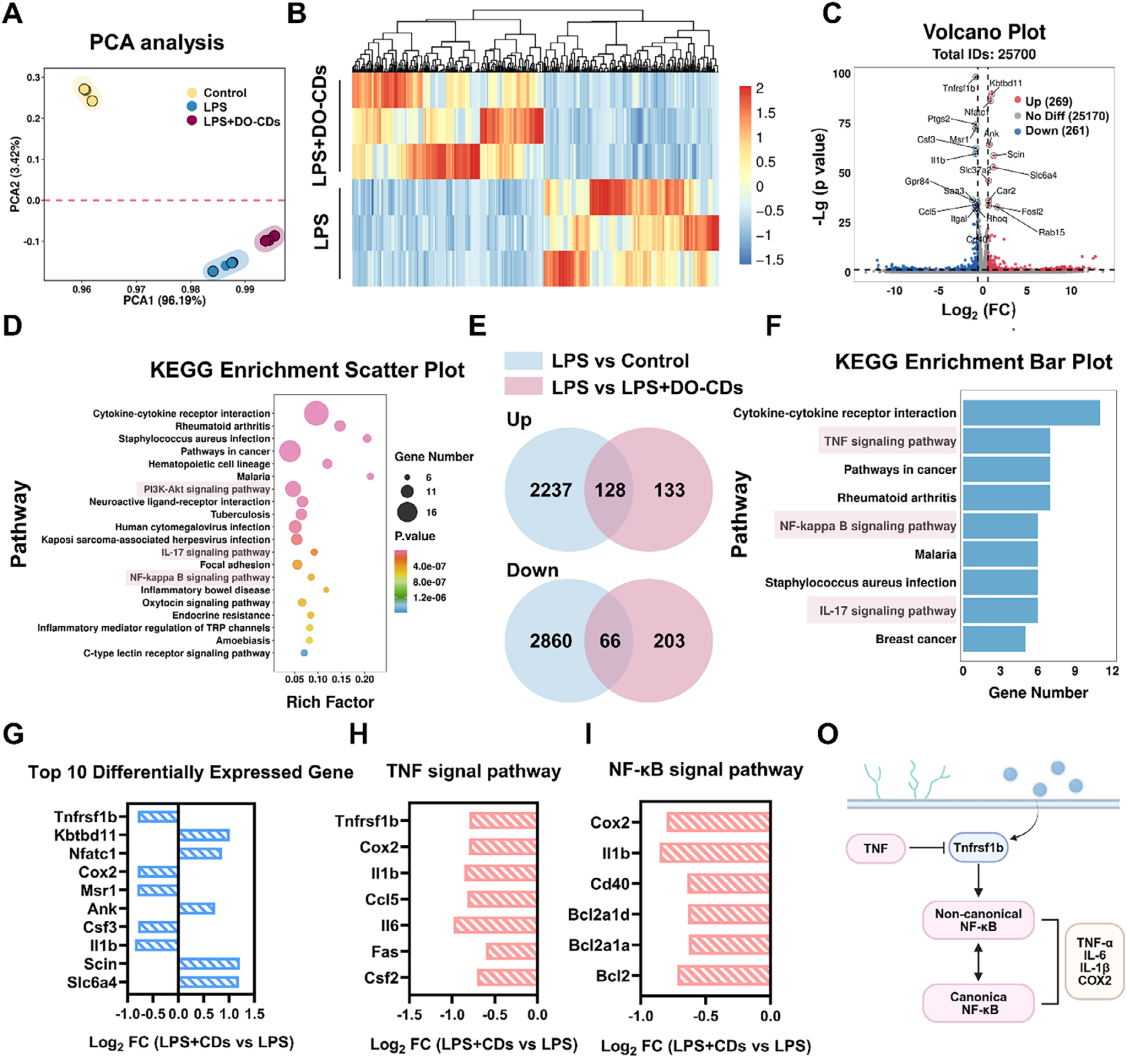
RNA‐Sequence analysis to reveal the biological mechanisms of DO‐CDs. A) PCA analysis of group “Control,” “LPS,” and “LPS + DO‐CDs.” The heatmap B) and volcano C) of the differentially expressed genes between group “LPS” and “LPS + DO‐CDs.” D) Analysis of KEGG enrichment. E) Venn diagram. F) KEGG enrichment analysis after filtering the differentially expressed genes. G) Top 10 differentially expressed genes between group “LPS” and “LPS + DO‐CDs.” (H) Differentially expressed genes in the TNF signal pathway. I) Differentially expressed genes in the NF‐κB signal pathway. O) Schematic of the biological mechanisms.

To explore how DO‐CDs exert anti‐inflammatory and antioxidant effects, we conducted transcriptome sequencing analysis on LPS‐stimulated macrophages. Principal component analysis showed significant characteristic differences among the three groups, which indicates the effectiveness of sample grouping and provides statistical evidence for the scientificity of subsequent analyses (**Figure** **5**A). Next, we compared the differentially expressed genes between the LPS group and the LPS + DO‐CDs group (Figure 5B). Compared with the LPS group, DO‐CDs upregulated 269 genes and downregulated 261 genes, which proves that DO‐CDs exert biological effects by regulating the expression of specific genes (Figure 5C). To further investigate the biological functions of these differentially expressed genes, we performed kyoto encyclopedia of gene and genomes (KEGG) enrichment analysis on the genes between the LPS group and LPS + DO‐CDs group, revealing that DO‐CDs might alleviate LPS‐induced cell damage through signaling pathways such as PI3K‐Akt, IL‐17, and NF‐κB. DO‐CDs may regulate the oxidative stress status of cells through these inflammatory pathways (Figure 5D). To more precisely elucidate the biological mechanism underlying DO‐CDs, we identified differentially expressed genes that were consistently upregulated or downregulated relative to the LPS group in both the Control group and the LPS + DO‐CDs group using a Venn diagram (Figure 5E). These genes indicated that DO‐CDs primarily function through the TNF‐NF‐κB signaling pathway (Figure 5F).

During colitis, inflammatory factors such as TNF‐α are released in large quantities.^[^ ^38^ ^]^ After TNF‐α binds to corresponding receptors on the cell surface, it activates the NF‐κB signaling pathway.^[^ ^39^ ^]^ As a critical transcription factor, NF‐κB translocates into the nucleus to trigger the transcription of inflammation‐associated genes.^[^ ^40^ ^]^ These inflammatory factors further exacerbate inflammatory responses, leading to intestinal tissue damage and sustained inflammation. The genes in the figures interact within the TNF‐NF‐κB signaling pathway, collectively influencing colitis progression (Figure 5G–I). Among them, genes including Cox2, IL‐1β, Ccl5, IL‐6, Csf2, and Cd40 promote inflammation by enhancing inflammatory responses,^[^ ^41^ ^]^ recruiting immune cells,^[^ ^42^ ^]^ and increasing inflammatory mediator production.^[^ ^43^ ^]^ Tnfrsf1b (tumor necrosis factor receptor superfamily member 1B),^[^ ^44^ ^]^ the gene with the most significant differential expression in transcriptome sequencing, mediates pro‐inflammatory effects primarily through the non‐canonical NF‐κB pathway.^[^ ^45^ ^]^ The cascading interaction between the canonical NF‐κB pathway and the non‐canonical NF‐κB pathway collectively constitutes the core signaling network of pro‐inflammation in colitis.^[^ ^46^ ^]^ Therefore, the potential therapeutic mechanism of DO‐CDs is as follows: by down‐regulating the expression of Tnfrsf1b, interfering with the upstream activation nodes of the non‐canonical NF‐κB pathway, thereby inhibiting the synergistic activation of the two pathways, and finally achieving a systematic reduction in inflammation levels (Figure 5O).

### 2.5 Bio‐Distribution of DO‐CDs In Vivo

**Figure 6:**
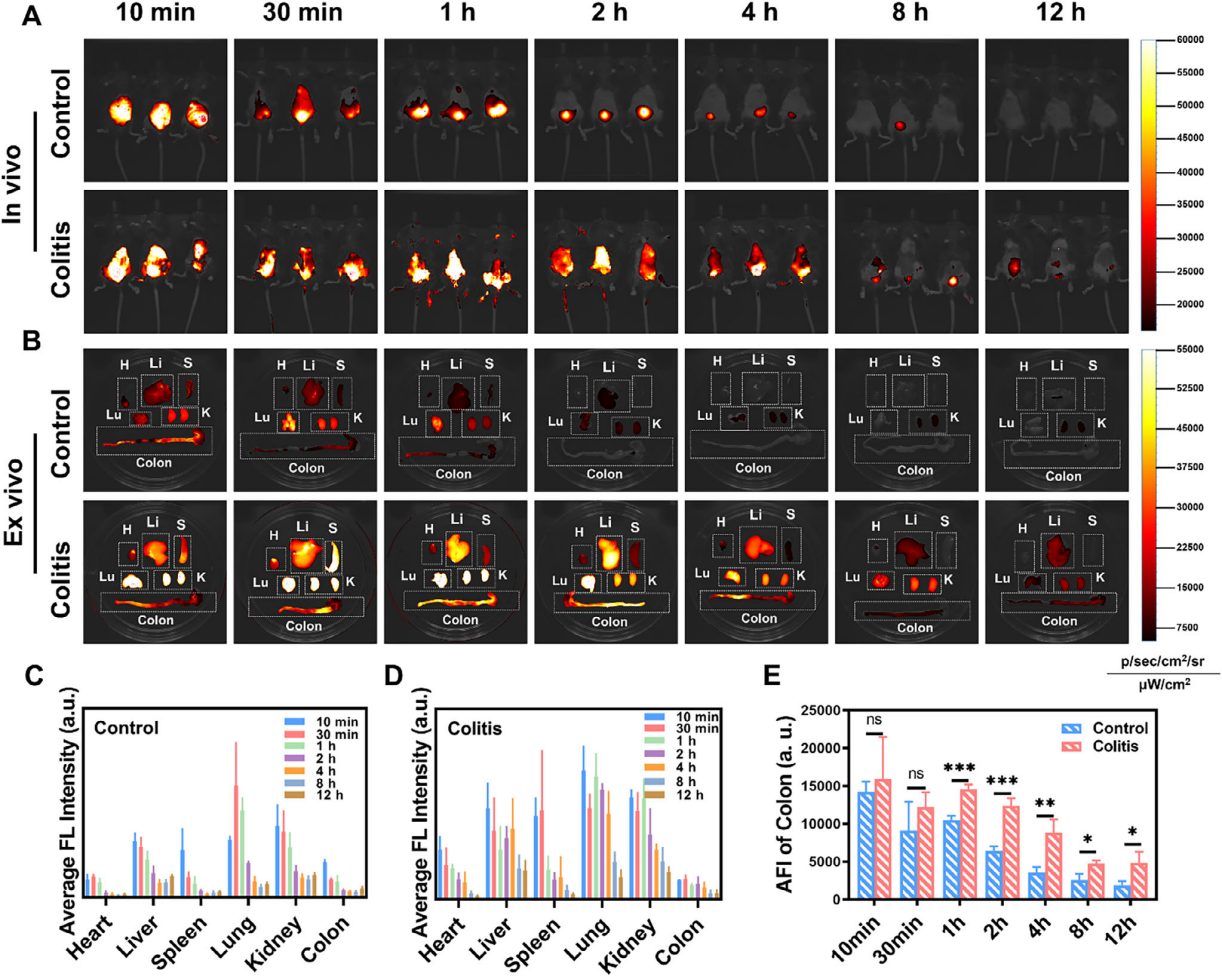
Bio‐distribution of DO‐CDs in vivo. A) Real‐time fluorescent signals in healthy versus colitis mice across different time points. B) Ex vivo organ imaging (H: heart, Li: liver, S: spleen, Lu: lung, K: kidney, colon) with corresponding groups. Quantitative analysis of organ‐specific accumulation in (C) healthy and D) diseased cohorts. E) Average fluorescence quantification in colonic tissue (*n* = 3). Statistical significance was indicated as ^*^ *p* < 0.05, ^**^ *p* < 0.01, ^***^ *p* < 0.001.

To systematically elucidate the distribution characteristics and metabolic patterns of DO‐CDs in vivo, this study covalently conjugated DO‐CDs with Cy5.5 as a red fluorescent marker, obtaining the near‐infrared fluorescent probe DO‐CDs‐Cy5.5. Both healthy mice and UC model mice received intraperitoneal injections of the probe. Near‐infrared (NIR) animal imaging was performed at multiple time points (10, 30 min, 1, 2, 4, 8, and 12 h postinjection) to dynamically monitor the spatiotemporal distribution of DO‐CDs‐Cy5.5 at whole‐body and organ levels (**Figure** **6**A,B). Experimental results demonstrated rapid absorption of DO‐CDs‐Cy5.5 via the superior mesenteric vein with specific accumulation in colonic tissues. In vivo imaging revealed peak abdominal fluorescence intensity at 10 min postinjection, followed by exponential decay, with significantly diminished signals by 12 h, indicating a short metabolic half‐life and near‐complete clearance within 12 h. Ex vivo imaging of major organs and colonic tissues at corresponding time points showed progressive fluorescence attenuation across all organs. The quantitative analysis identified significantly higher fluorescence intensity in the liver and kidneys, the primary filtration organs, compared to other tissues, suggesting renal dominance in DO‐CD excretion (Figure 6C,D). Given the pathophysiological disparities between healthy and UC mice, comparative analysis revealed substantially enhanced fluorescence intensity in UC model mice (Figure 6E). In vivo, imaging studies of DO‐CDs demonstrated their rapid targeted accumulation at inflamed sites, followed by efficient short‐term clearance. This characteristic provides essential pharmacokinetic evidence for subsequent therapeutic experiments in animal models and significantly supports the potential translation of precision therapy for IBD.

### 2.6 Therapeutic Effects of DO‐CDs on Acute Colitis

**Figure 7:**
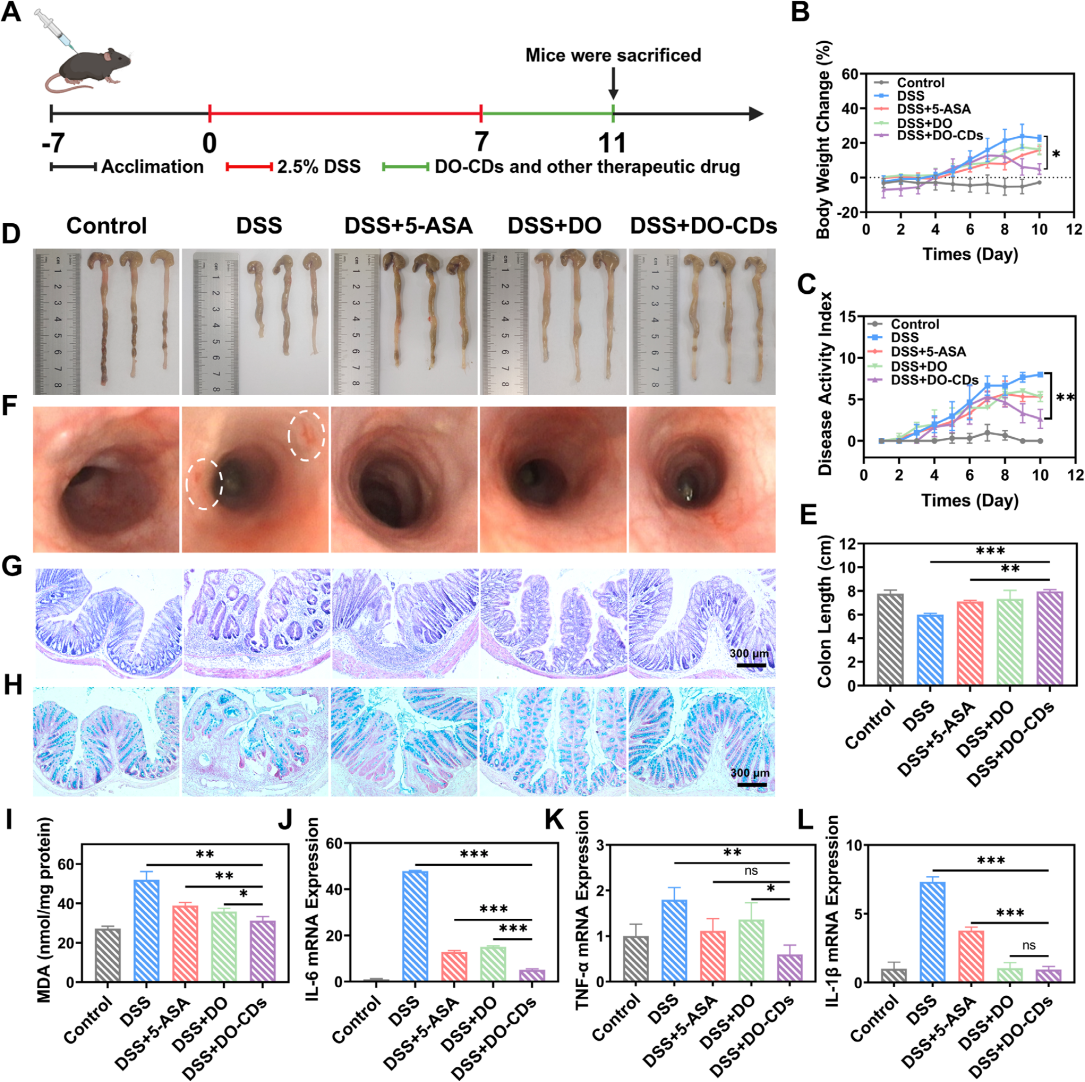
Therapeutic effects of DO‐CDs on acute colitis. A) Schematic representation of the experimental design. B) Body weight monitoring. C) DAI scores. Colon photographs D) and length statistics E). F) Intestinal endoscopic images. H&E staining G) and Alcian blue staining H) of colon pathological sections (scale bar: 300 µm). I) MDA content in colon tissues. J–L) qPCR quantification of pro‐inflammatory mediators (IL‐6/TNF ‐ α/IL ‐ 1β) in colons. (*n* = 3). Statistical significance was indicated as ^*^ *p* < 0.05, ^**^ *p* < 0.01, ^***^ *p* < 0.001.

To comprehensively investigate the therapeutic efficacy of DO‐CDs on colitis at the animal level, this study established an acute colitis model by administering 2.5% DSS in drinking water to mice for 7 consecutive days. Following successful model induction, a 4‐day therapeutic intervention was implemented with daily monitoring of body weight changes, fecal characteristics, and Disease Activity Index (DAI) (**Figure** **7**A). Typical pathological manifestations of colitis, including decreased mobility, significant weight loss with a 5% reduction in body weight, a ≈20% shortening of colonic length, and hematochezia, emerged progressively from day 5 after modeling. Postmodeling, mice were randomized into five groups based on weight changes: Control, DSS, DSS + 5‐ASA, DSS + DO, and DSS + DO‐CDs.

Tissue collection revealed that pharmacological interventions induced body weight recovery, with the DSS + DO‐CDs group exhibiting the most pronounced restoration (Figure 7B), Meanwhile, the disease activity index (DAI) score of the DSS+DO‐CDs group showed a synchronous downward trend, indicating that the vitality of mice was gradually recovering with the improvement of body weight and inflammatory status (Figure 7C). Concurrently, colon length measurements demonstrated statistically significant differences between DSS + DO‐CDs and DSS groups (Figure 7D). It indicated that DO‐CDs restored the length of the colon (Figure 7E). Endoscopic evaluation showed marked inflammation and hemorrhagic lesions in DSS controls, while therapeutic groups displayed improved vascular clarity and reduced bleeding (Figure 7F). Histopathological analysis via hematoxylin‐eosin (H&E) and alcian blue staining identified severe inflammatory cell infiltration, crypt architecture destruction, and goblet cell depletion in DSS‐treated colons. Therapeutic groups exhibited restored intestinal barrier integrity (Figure 7G,H). Given the pivotal role of ROS in colitis pathogenesis, we quantified malondialdehyde (MDA)‐ a lipid peroxidation end product that initiates membrane dysfunction through polyunsaturated fatty acid oxidation in colonic mucosa. DO‐CDs treatment significantly reduced MDA levels, indicating effective mitigation of oxidative stress (Figure 7I). qPCR analysis of colon tissue RNA further demonstrated DO‐CDs‐mediated downregulation of pro‐inflammatory cytokines TNF‐α, IL‐6, and IL‐1β (Figure 7J–L), indicating DO‐CDs effectively alleviate intestinal inflammation. Collectively, these findings substantiate that DO‐CDs effectively restore intestinal barrier function and ameliorate acute colitis progression through dual mechanisms of oxidative stress suppression and inflammatory cytokine modulation.

### 2.7 The Therapeutic Effect of DO‐CDs on Chronic Colitis

**Figure 8:**
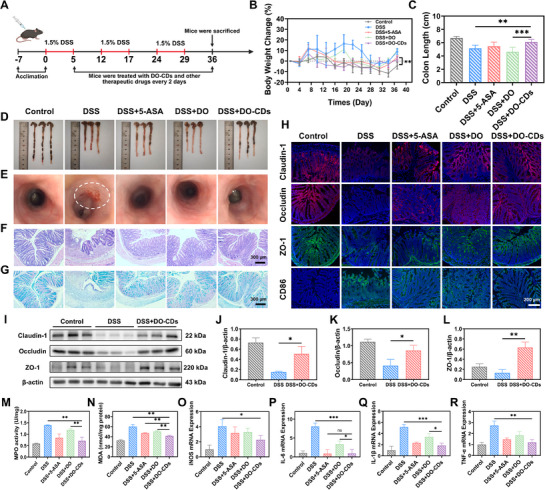
Therapeutic effects of DO‐CDs on chronic colitis. A) Schematic illustration of the experimental scheme for assessing DO‐CDs’ therapeutic impacts in chronic colitis models. B) Body weight fluctuations. Colon images D) and length measurements C). E) Intestinal endoscopic views. F) H&E staining. G) Alcian blue staining (scale bar: 300 µm). H) Immunofluorescence images of claudin ‐ 1, occludin, ZO‐1, and CD86 in colons (scale bar: 200 µm). Western blot analysis I) and quantitative graphs for claudin ‐1 J), occludin K), and ZO‐1 L). MPO activity M) and MDA content N) in colon tissues. mRNA expression levels of iNOS O), IL‐6 P), IL‐1β Q), and TNF‐α R). (*n* = 3). Statistical significance was indicated as ^*^ *p* < 0.05, ^**^ *p* < 0.01, ^***^ *p* < 0.001.

To investigate the therapeutic efficacy of DO‐CDs on chronic colitis characterized by persistent and recurrent inflammation, a chronic colitis mouse model was established to better simulate the pathological progression (**Figure** **8**A). The data showed significant weight loss in mice during the DSS‐feeding phase. Although partial weight recovery occurred in the recovery period, the DSS group exhibited markedly impeded weight restoration after the second round of DSS modeling due to colitis recurrence. In contrast, the DSS + DO‐CDs group demonstrated superior weight recovery (Figure 8B). Figure 8C,D illustrates the colon morphology across various groups, revealing a statistically significant increase in colon length in the DSS + DO‐CDs group compared to the DSS group. Endoscopic examination revealed severe intestinal wall bleeding, tissue fragility, and loss of elasticity in the DSS group caused by chronic recurrent injury, whereas DO‐CDs treatment effectively alleviated intestinal hemorrhage (Figure 8E). H&E and alcian blue staining demonstrated more intact crypt structures and higher mucin content in the DSS + DO‐CDs group than in the DSS group, indicating mucosal barrier repair (Figure 8F,G).

Intestinal tight junction proteins are located between intestinal epithelial cells, forming a “zipper”‐like closed structure and serving as a key component of the intestinal epithelial barrier.^[^ ^47^ ^]^ When the expression of tight junction proteins is reduced or their structure is disrupted, intestinal epithelial permeability increases, allowing harmful substances to easily enter the bloodstream and trigger systemic inflammation.^[^ ^48^ ^]^ Immunofluorescence staining and Western blot analysis of colonic tight junction proteins revealed a significant upregulation in the expression of Occludin, ZO‐1, and Claudin‐1 following DO‐CDs treatment. This upregulation was associated with a reduction in the translocation of deleterious substances from the intestinal lumen to the lamina propria, consequently decreasing the activation frequency of immune cells within the lamina propria. These findings suggest that the epithelial barrier of the colon was effectively repaired (Figure 8H–L).

Because excessive activation of M1 macrophages is closely associated with immune cell infiltration in the intestinal wall, crypt abscess formation, and mucosal barrier damage, the significant reduction of CD86‐labeled M1 macrophages in the DSS + DO‐CDs group suggests that DO‐CDs exhibit a favorable repairing effect on the physical barrier damage caused by colitis (Figure 8H). Myeloperoxidase (MPO), a marker enzyme of neutrophil activation whose activity positively correlates with neutrophil infiltration at inflammatory sites, showed decreased levels in the DSS + DO‐CDs group, indicating reduced neutrophil migration and attenuated local inflammation (Figure 8M). Malondialdehyde (MDA), the end product of lipid peroxidation reflecting oxidative damage to biomembranes by free radicals, exhibited reduced levels in DSS + DO‐CDs group colons, demonstrating improved oxidative stress status (Figure 8N). For inflammation assessment, qPCR data confirmed that DO‐CDs significantly downregulated the expression of pro‐inflammatory cytokines iNOS, IL‐6, IL‐1β, and TNF‐α (Figure 8O–R). These findings indicate that DO‐CDs treatment is effective in re‐establishing the integrity of mucosal and epithelial barriers in cases of chronic colitis.

### 2.8 The Effects of DO‐CDs on the Intestinal Immune Barrier

**Figure 9:**
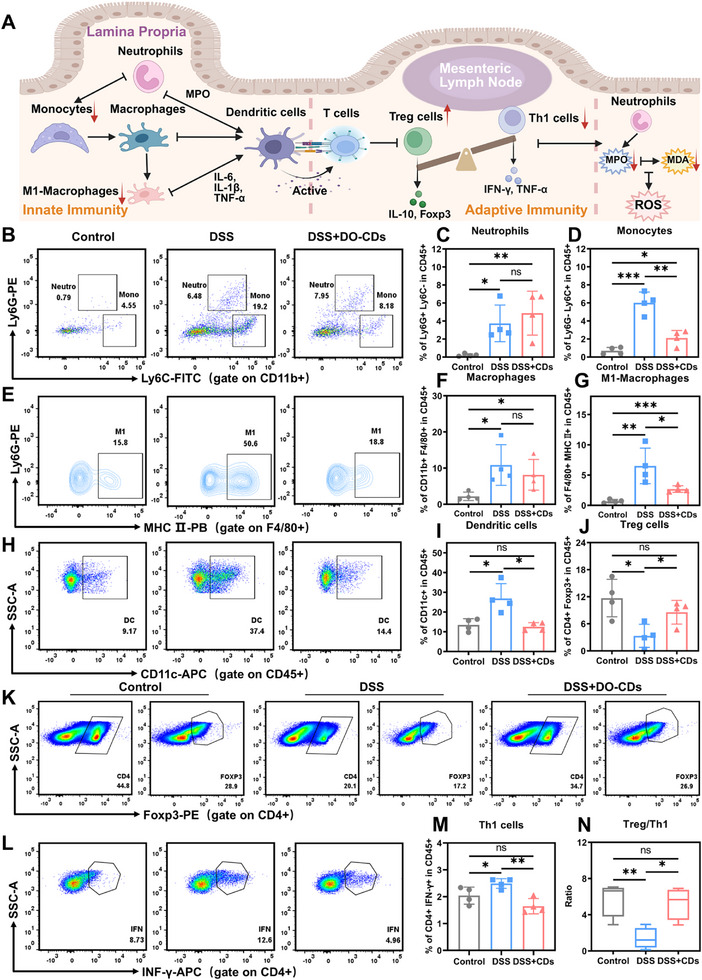
The effects of DO‐CDs on immune cells in chronic colitis. A) Schematic diagram of the effects of DO‐CDs on innate and adaptive immunity. B) Flow cytometric examination of the neutrophils and monocytes. Statistics of neutrophils C) and monocytes D). E) Flow cytometric examination of the M1‐phenotype macrophages. Statistics of macrophages F) and M1‐phenotype macrophages G). H) Flow cytometric examination of the dendritic cell. Statistics of dendritic cell I) and Treg cells J). K) Flow cytometric examination of the Treg cells. L) Flow cytometric examination of the Th1 cells. Statistics of Th1 cells M). N) The ratio of Treg cells and Th1 cells. (*n* = 4). Statistical significance was indicated as ^*^ *p* < 0.05, ^**^ *p* < 0.01, ^***^ *p* < 0.001.

The intestinal immune barrier, serving as a critical defense against pathogen invasion, plays a central role in colitis pathogenesis.^[^ ^49^ ^]^ This barrier not only precisely identifies and eliminates foreign pathogens such as bacteria and viruses but also initiates epithelial repair programs through cytokine and growth factor release from immune cells during mucosal damage, thereby maintaining intestinal homeostasis.^[^ ^50^ ^]^ Composed of innate and adaptive immune systems, the intestinal immune barrier operates through coordinated mechanisms: innate immunity relies on rapid responses from neutrophils, macrophages, and dendritic cells, while adaptive immunity regulates immune responses via T cell subsets following dendritic cell‐mediated antigen presentation. The dynamic equilibrium between these systems underpins intestinal immune homeostasis, as illustrated by the immune cell interaction network in **Figure** **9**A.

To investigate the immunomodulatory mechanism of DO‐CDs in chronic colitis, colonic immune cells were collected immediately after the third DSS cycle to simulate clinical scenarios with concurrent drug intervention and colitis recurrence. Using the flow cytometry gating strategy in Figures S3–S6 (Supporting Information), we analyzed the proportional and numerical changes of immune cell subsets across treatment groups (Figures S3–S6, Supporting Information). Flow cytometry analysis revealed no significant difference in lamina propria neutrophil proportions between DSS and DSS + DO‐CDs groups (Figure 9B,C), attributed to persistent inflammation‐driven neutrophil recruitment dynamics and their short lifespan, maintaining a “continuous recruitment‐rapid apoptosis” equilibrium. Conversely, our observations indicated a significant reduction in the proportion of monocytes following treatment with DO‐CDs.Monocytes can differentiate into macrophages and dendritic cells. Their reduction indicates that DO‐CDs are effectively suppressing immune responses, thereby controlling inflammatory activity and stabilizing immune responses (Figure 9D). Although macrophage proportions in the DSS + DO‐CDs group did not reach statistical significance, they exhibited a downward trend (Figure 9E,F), with pro‐inflammatory M1‐macrophages and dendritic cells being significantly reduced compared to the DSS group (Figure 9G). These results indicate that DO‐CDs may interrupt the inflammatory cascade by regulating macrophage polarization at the level of innate immunity and inhibiting the excessive activation of dendritic cells, thereby reducing antigen presentation, suppressing abnormal T cell activation, and further inhibiting subsequent adaptive immune changes (Figure 9H,I).

Adaptive immunity is activated following antigen presentation by dendritic cells (DCs).^[^ ^51^ ^]^ We assessed the proportions of Th1 and Treg cells in mesenteric lymph nodes using flow cytometry. Th1 cells primarily maintain chronic intestinal inflammation by secreting pro‐inflammatory cytokines dominated by IFN‐γ and TNF‐α. Among these, IFN‐γ reinforces the pro‐inflammatory phenotype of innate immunity through cell‐cell interactions, enhances antigen‐presenting capacity, and promotes the recruitment of immune cells to inflamed sites.^[^ ^52^ ^]^ Treg cells, conversely, are central regulators of intestinal immune homeostasis in colitis; their deficiency directly leads to imbalanced immune tolerance and mucosal barrier damage.^[^ ^53^ ^]^ Statistical results showed that compared with the DSS group, the DSS + DO‐CDs group exhibited a significant reduction in Th1 cell proportion, an increase in Treg cell numbers, and restoration of the Treg/Th1 ratio to control levels (Figure 9J–N). These findings indicate that DO‐CDs reverse the pathological processes of macrophage polarization to the pro‐inflammatory M1 phenotype, exacerbate intestinal mucosal inflammatory infiltration, and enhance oxidative damage caused by excessive Th1 cell activation by bidirectionally regulating the dynamic balance of Th1/Treg cells. This effect confirms the therapeutic ability of DO‐CDs to correct immune dysregulation and provides direct immunological evidence for their role in mucosal barrier repair in chronic colitis. Significantly, the restoration of the Th1/Treg balance not only interrupts the positive feedback loop of “Th1 cell activation‐M1 macrophage polarization‐inflammation amplification” but also synergistically facilitates the development of an anti‐inflammatory microenvironment and the repair of the intestinal epithelium by reinstating the immunosuppressive functions of Treg cells. This underscores the synergistic mechanism between the immunoregulatory and barrier‐reparative effects of DO‐CDs.

### 2.9 The Biocompatibility of DO‐CDs

**Figure 10:**
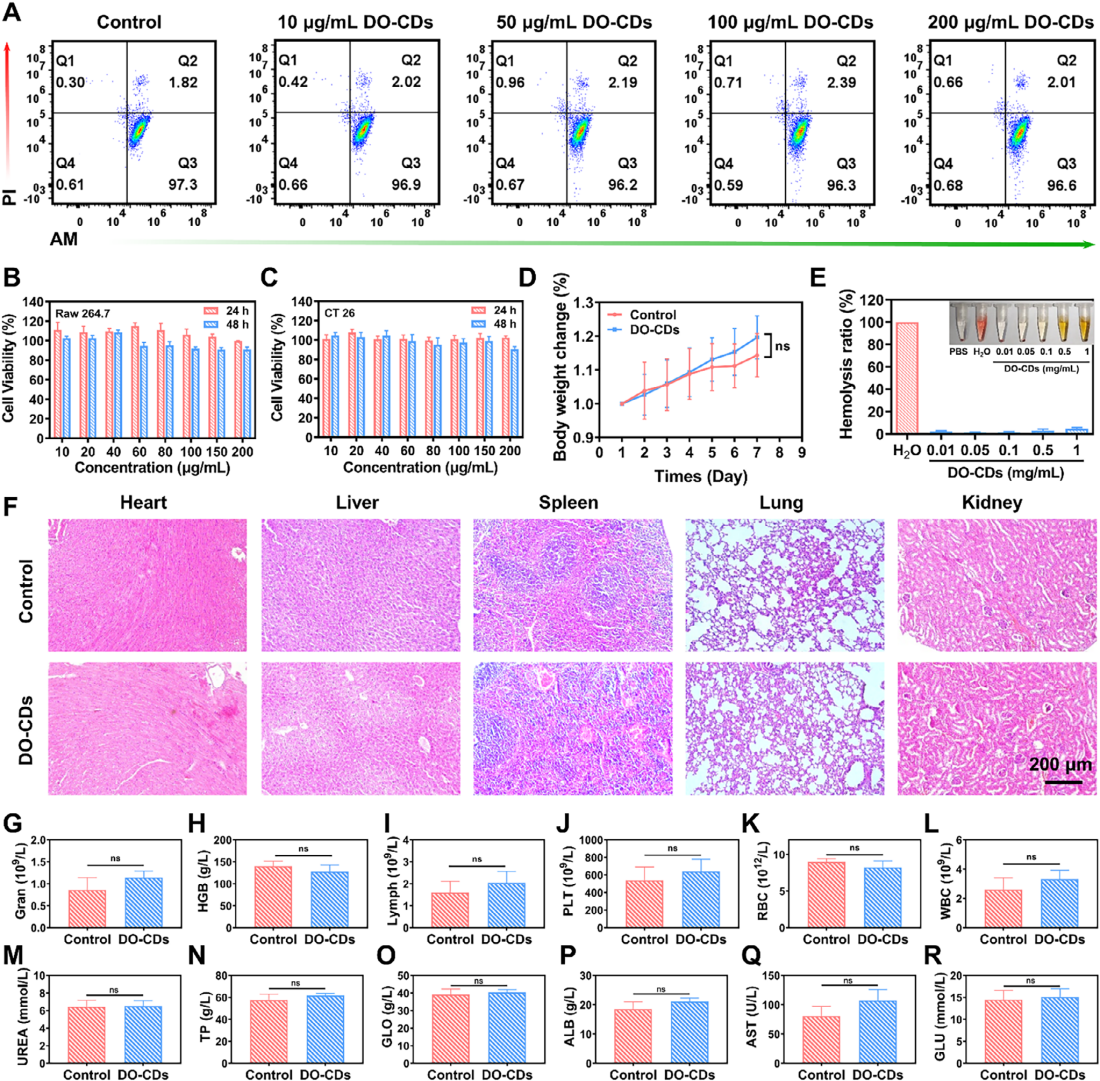
Biocompatibility of DO‐CDs. A) Flow cytometry analysis of viable RAW264.7 cells. Viability of RAW264.7 B) and CT26 C) cells following 12‐ and 24‐h DO‐CDs exposure. D) Body weight dynamics in DO‐CDs‐treated mice. E) Hemolysis assay results. F) H&E staining of major organs (heart, liver, spleen, lung, kidney) (scale bar: 200 µm). G–R) Blood biochemical and hematological parameters. (*n* = 3). Statistical significance was indicated as ^*^ *p* < 0.05, ^**^ *p* < 0.01, ^***^ *p* < 0.001.

Biocompatibility serves as the fundamental basis for nanomaterials to exert biological functions. To assess the potential toxicity of DO‐CDs on various tissues and organs, we evaluated the biocompatibility of DO‐CDs at both cellular and animal levels. The cytotoxicity of DO‐CDs was verified through live/dead cell staining assays. Calcein‐AM, a nonfluorescent substrate, emits green fluorescence upon cleavage by intracellular esterases into polar calcein retained in viable cells, whereas dead cells lacking esterase activity are labeled red by propidium iodide (PI). Flow cytometry demonstrated a 96.6% viable cell ratio in RAW264.7 cells after 24‐h incubation with 200 µg mL^−1^ DO‐CDs (**Figure** **10**A). MTT assays confirmed in vitro biosafety, showing >80% viability in both RAW264.7 and CT26 cells after 24‐ and 48‐h exposure to 200 µg mL^−1^ DO‐CDs (Figure 10B,C).

At the animal level, systemic biocompatibility was evaluated by daily intraperitoneal injection of 20 mg kg^−1^ DO‐CDs over 7 days, with body weight monitored throughout the treatment. Post‐treatment collection of major organs and blood samples enabled comprehensive analysis. (Figure 10D). Hemocompatibility testing revealed a hemolysis rate of only 4.78% after 4‐h incubation of erythrocytes with 1 mg mL^−1^ DO‐CDs at 37 °C (Figure 10E). Histopathological evaluation by H&E staining confirmed no observable organ damage in DO‐CDs‐treated mice (Figure 10F). Comparative analysis of routine blood parameters and organ function biomarkers showed no statistically significant differences between groups (Figure 10G–R). In addition, the post‐treatment blood test data indicate that while effectively treating acute colitis, DO‐CDs can improve inflammation‐induced abnormalities in blood indices and metabolic disorders, without causing acute damage to important organs such as the liver and kidneys (Figure S7, Supporting Information). These collective data demonstrate the favorable biocompatibility of DO‐CDs.

## 3 Conclusion

In summary, our study systematically evaluated the therapeutic potential of DO‐CDs through ROS scavenging, inflammatory regulation, and intestinal barrier restoration in both in vivo and in vitro models. In the DSS‐induced chronic colitis model, DO‐CDs effectively reduced the proportion of M1‐macrophages, inhibited dendritic cell activation, restored the Th1/Treg cell balance, and downregulated pro‐inflammatory cytokine expression. Due to reduced inflammatory cell infiltration, increased expression of tight junction proteins between colonic epithelial cells facilitated the repair of the intestinal mechanical barrier. Concurrently, mucins secreted by goblet cells formed a protective mucus layer over the epithelial barrier. The restored mucosal barrier effectively isolated and controlled microbial colonization and invasion. These results demonstrate that DO‐CDs alleviate colitis progression by remodeling the mucosal‐mechanical‐immune barrier triad, thereby providing a theoretical foundation and technical reference for the biomedical application of natural product‐derived CDs. Future investigations should focus on elucidating long‐term toxicity, optimizing pharmacokinetic profiles, and developing targeted delivery strategies to advance their clinical translation for inflammatory disease therapy.

## 4 Experimental Section

### Materials

*Dendrobium officinale* was commercially available from *Tongxinzibu* health‐preserving medicinal materials. ABTS assay kit, DAPI staining solution, JC‐1 detection kit, RNAeasy Animal RNA Isolation Kit with Spin Column, and dihydroethidium (DHE) probe were purchased from Beyotime (Shanghai, China). The SOD detection kit (WST method) was obtained from Dojindo (Shanghai, China). The 2′‐7′‐Dichlorodihydrofluorescein (DCFH) probe, Triton X‐100, TMB, and MTT were commercially acquired from Solarbio (Beijing, China). Lipopolysaccharide (LPS) and dextran sulfate sodium (DSS) were procured from Sigma‐Aldrich and MP Biomedicals, respectively. Commercial myeloperoxidase (MPO) and malondialdehyde (MDA) detection kits originated from Nanjing Jiancheng Bioengineering Institute. 1‐ethyl‐3‐(3‐dimethylaminopropyl) carbodiimide (EDC) and N‐hydroxysuccinimide (NHS) were purchased from Aladdin Reagent (Shanghai, China); Cy5.5 fluorescent dye was purchased from Xi'an Ruixi Biotechnology. All aqueous solutions were prepared using deionized water. Antibodies (Biolegend, Table S1, Supporting Information).

### Preparation of DO‐CDs

The dried *Dendrobium officinale* was crushed into a powder. Then, the powder was uniformly dispersed in ultrapure water to form a homogeneous dispersion system. The mixture was transferred into a sealed high‐pressure autoclave and subjected to a hydrothermal reaction at a constant temperature of 180 °C for 12 h. After the reaction was completed, the obtained solution was filtered through a membrane with a pore size of 0.22 µm to remove insoluble impurities. Subsequently, purification was performed via dialysis for 24 h using a 1000 Da molecular weight cut‐off dialysis bag to further separate and eliminate small molecular impurities. Finally, the DO‐CDs were obtained through freeze‐drying.

### Preparation of DO‐Extract

An equal mass of *Dendrobium Officinale* medicinal material, as the raw material used in the synthesis of DO‐CDs was weighed, and an equal volume of ultrapure water, as that in the carbon dot synthesis system, was added. The mixture was boiled over high heat in an earthen pot, then turned to low heat and decocted for 30–40 min. After decoction, the medicinal solution was first filtered through gauze to remove residues and then further purified using a 0.22 µm microporous membrane. The collected filtrate was processed by freeze‐drying technology, and finally, the dried *Dendrobium Officinale* extract was obtained for subsequent control experiments.

### Characterization of DO‐CDs

Morphological characterization of DO‐CDs was conducted via transmission electron microscopy (TEM) and atomic force microscopy (AFM). Malvern Zetasizer Nano ZSE was used to measure the zeta potential. Using SHIMADZU UV‐2700 and HITACHI F‐4700 systems to measure UV–vis absorption and fluorescence spectra. FT‐IR and XPS analyses employed the Nicolet 5700 (Thermo Scientific) and ESCALAB Xi+ (Thermo Fisher) spectrometers. In vivo, murine tracking and ex vivo tissue imaging utilized the VISQUE InVivo Smart‐LF multimodal platform. Analytical services were provided by Beijing Zhongkebaice Technology Service Co., Ltd. (www.zkbaice.cn). The free radical signals of the samples were measured with an electronic paramagnetic resonance spectroscope (EPR, CIQTEK EPR200‐pLUS).

### Synthesize DO‐CDs‐cy5.5

First, EDC (10 mg mL^−1^, 100 µL) and NHS (10 mg mL^−1^, 200 µL) were reacted at 25 °C for 30 min to activate functional groups. Second, DO‐CDs (5 mg mL^−1^, 1 mL) were incorporated into the solution and stirred under light‐protected conditions at room temperature for 1 h. Finally, Cy5.5 (0.25 mg mL^−1^, 2 mL) was introduced, and the mixture was continuously stirred in the dark at ambient temperature for 24 h. The resulting solution was dialyzed using a 1000 Da molecular weight cutoff (MWCO) membrane for 24 h to obtain red‐fluorescent DO‐CDs‐Cy5.5.

### Cellular Uptake and Co‐Localization

DO‐CDs‐cy5.5 were utilized to investigate cellular uptake and co‐localization. RAW264.7 cells were seeded onto cell culture slides and allowed to adhere and grow for 24 h. Subsequently, the cells were incubated with DO‐CDs‐Cy5.5 for 2, 4, and 6 h, respectively. Following the incubation periods, quantitative analysis was performed using a flow cytometer, and fluorescence microscopy images were captured to visualize the cellular internalization of DO‐CDs‐Cy5.5. To explore the subcellular localization of DO‐CDs‐Cy5.5, mitochondria were labeled with Mito‐Tracker Green, while lysosomes were stained with Lyso‐Tracker Green. The co‐localization of DO‐CDs‐Cy5.5 with mitochondria and lysosomes was observed and imaged using an upright microscope. The acquired fluorescence microscopy images were further processed and analyzed by using Image‐J.

### Intracellular Antioxidant Effects of DO‐CDs

RAW264.7 cells were seeded in cell culture plates and allowed to adhere and stabilize overnight. Subsequently, the cells were co‐incubated with DO‐CDs for a period of 5 h. To induce oxidative stress within the cells, hydrogen peroxide (H_2_O_2_) was added to the culture medium at a final concentration of 600 µm for 2 h. To evaluate the intracellular ROS scavenging capacity of DO‐CDs, DCFH and DHE were employed as fluorescent probes. After the completion of cell staining procedures, quantitative analysis of ROS levels was performed using a flow cytometer, and fluorescence microscopy images were captured to visually assess the distribution and intensity of ROS signals within the cells, thereby enabling a comprehensive evaluation of the protective effects of DO ‐ CDs against oxidative stress in RAW264.7 cells.

### Intracellular Anti‐Inflammatory Effects of DO‐CDs

RAW264.7 macrophages were plated overnight, followed by 5‐h DO‐CD exposure. LPS (1 mg mL^−1^) was then introduced into the medium for 12‐h inflammatory stimulation. Poststimulation, CD86 surface staining (30 min) preceded flow cytometric quantification of macrophage polarization. Parallel qPCR analyses precisely measured pro‐/anti‐inflammatory cytokine mRNA levels.

### JC‐1 Staining

RAW264.7 cells preseeded overnight were exposed to DO‐CDs for 5 h, followed by 4 h of 500 µm H_2_O_2_ treatment to induce mitochondrial impairment. JC‐1‐stained samples underwent fluorescence imaging after 30 min incubation.

### Animals

C57BL/6 female mice (8 weeks old) were supplied by the Medical Experimental Animal Center of Xi'an Jiaotong University, Shaanxi Province, China. All animal experiments were conducted following the Guidelines for the Care and Use of Laboratory Animals of Xi'an Jiaotong University and approved by the Animal Ethics Committee.

### In Vivo Animal Image

DO‐CDs‐Cy5.5 conjugates were utilized for systemic distribution profiling. C57BL/6 mice were randomized into control and colitis cohorts. Acute colitis was induced by supplementing drinking water with 2.5% DSS for 7 consecutive days. Real‐time in vivo fluorescence tracking and *ex vivo* organ imaging were performed using the VISQUE InVivo imaging system at 10 min, 30 min, 1, 2, 4, 8, and 12 h after intraperitoneal injection.

### Biosafety Evaluation

RAW264.7 and CT26 cells were plated and allowed to adhere overnight, then co‐cultured with DO‐CDs at varying concentrations for 24 or 48 h. The MTT assay was used to assess cell viability. Mice were randomly divided into two groups: Control and DO‐CDs. The DO‐CDs group received daily intraperitoneal injections of 20 mg kg^−1^ DO‐CDs for 7 days, while the control group remained untreated. Seven days post‐treatment, mice were euthanized, and biocompatibility was evaluated via pathological analyses of major organs, routine blood test results, and blood biochemical parameters.

### DSS‐Induced Colitis of Mice

Acute colitis: C57BL/6 mice (6–8 weeks old) were employed to generate acute colitis models. Animals were randomized into groups (≥6 mice/group) and subjected to 2.5% DSS administration in drinking water for 7 consecutive days following 1‐week acclimatization to induce colitis. After 7 days, the mice received different treatments: 20 mg kg^−1^ DO‐CDs, 20 mg kg^−1^ DO, or 50 mg kg^−1^ 5‐ASA.

### Chronic Colitis

Chronic colitis models were established using 6–8‐week‐old C57BL/6 mice. Animals were randomized into groups (≥6/group) and acclimatized for 1 week before subsequent procedures. The mice's drinking water was replaced with an aqueous solution containing 1.5 % DSS, and they were continuously fed with this solution for 5 days. Subsequently, they were switched to pure water for 7 days, and this cycle was repeated three times. Beginning from the second round of DSS feeding, the mice were administered the drug once every two days (20 mg kg^−1^ of DO‐CDs, 20 mg kg^−1^ of DO, 50 mg kg^−1^ of 5‐ASA). Meanwhile, their weight fluctuations, fecal characteristics, and activity status were meticulously recorded daily. After the completion of the third round of model establishment, the mice were sacrificed, and their colonic tissues were collected for subsequent in‐depth analysis.

### Western Blotting

Colonic tissues were lysed in RIPA buffer (Abclonal) supplemented with 1× protease/phosphatase inhibitor cocktail (NCM Biotech) on ice for 30 min. Protein separation was performed using 10% PAGE gels (Epizyme Biomedical Technology), followed by transfer to 0.22 µm PVDF membranes. Membranes were blocked with 5% nonfat milk and incubated overnight with primary antibodies: anticlaudin‐1 (Proteintech, 13050‐1‐AP), anti‐ZO‐1 (Proteintech, CL488‐21773), and antioccludin (Proteintech, CL594‐13409). After washing with TBST containing 2% Tween, membranes were incubated with HRP‐conjugated secondary antibodies (Proteintech) for 2 h. Chemiluminescent signals were detected using the ChemiScope 6100 imaging system.

### Immunoflow Cytometry

Mice were euthanized via cervical dislocation, and colons were excised into a complete HBSS solution. Intestinal segments were longitudinally incised with surgical scissors, washed with complete HBSS to remove residual luminal contents, vortexed in complete HBSS for 1 min, and mechanically dissected. Tissues were enzymatically digested with 1 mg mL^−1^ collagenase IV (Sigma) and 0.2 mg mL^−1^ DNase I (Beyotime) at 37 °C for 1 h. Digested samples were filtered through a 100 µm nylon mesh into PBS supplemented with 2% FBS, followed by repeated centrifugation and washing. Lymph nodes were mechanically homogenized before analysis.

For cell surface staining, take 100 µL of the cell suspension (≈2 × 10^6^ cells). First, perform anti‐FcR (Bioxcell, 2.4G2) blocking on the membrane surface for each sample. Add 10 µL of the antibody mixture (all 1: 200) for surface antibody staining. Stain at 4 °C in the dark for 30 min. After staining, add 2 mL of staining buffer, wash 1–2 times, centrifuge at 1900 rpm for 5 min, discard the supernatant, and resuspend 200 µL of staining buffer, ready for machine detection.

For intracytoplasmic staining, cells (3 × 10⁵) were incubated in 48‐well plates containing complete RPMI‐1640 medium (Gibco) supplemented with Cell Stimulation and Protein Transport Inhibitor Cocktails (eBioscience). Five hours later, cells were collected and processed using the BioLegend Cytokine Staining Kit protocol. Surface staining was performed first, followed by the addition of 2 mL staining buffer. After 1‐2 washes with centrifugation at 1900 rpm for 5 min, the supernatant was removed. Cells were fixed with 80 µL BD Fixation Buffer (20 min, 4 °C) and washed twice with 150 µL BD 1×Perm Wash Buffer. Antibodies were diluted in 1×Perm Wash Buffer for 30‐min intracellular staining at 4 °C. Poststaining procedures included 1‐2 washes (1900 rpm, 5 min) and final resuspension in 200 µL 1×Perm Wash Buffer for detection. Experimental antibodies are detailed in Table S1 (Supporting Information). FlowJo software facilitated data analysis.

### Statistical Analysis

All data were analyzed using GraphPad Prism 9.0 software. Results are presented as mean ± SD from three separate experiments. One‐way ANOVA and t‐test were employed for statistical analyses, with significance levels denoted as ^*^ *p* < 0.05, ^**^ *p* < 0.01, and ^***^ *p* < 0.001.

## Conflict of Interest

The authors declare no conflict of interest.

## Author Contributions

C.X. and X.H. contribute equally to this work. C.X. performed the investigation, methodology, and wrote the original draft. X.H. performed methodology, data curation. Z.D. performed conceptualization, methodology, and formal analysis. R.W. performed formal analysis. S.G. and J.F. performed the methodology. L.Y. performed the investigation, data curation. Y.Z. performed data curation. B.G. Wrote, reviewed, and edited. M.Z. Wrote, reviewed, and edited the final draft, resources, supervision, and funding acquisition. X.Z. performed supervision, funding acquisition. M.Y. performed supervision, funding acquisition. M.Z. performed conceptualization, Wrote, reviewed, and edited, resources, supervision, funding acquisition, and project administration.

## Supporting information



Supporting Information

## Data Availability

The data that support the findings of this study are available from the corresponding author upon reasonable request.
