# Supplementary material for: Dendrobium Officinale‐Derived Carbon Dots Nanozymes Alleviate Colitis by Orchestrating Intestinal Mucus‐Epithelium‐Immune Barriers
Source: Adv Sci (Weinh). 2025 Oct 17;12(44):e12567. doi: 10.1002/advs.202512567 (PMC12667543; doi:10.1002/advs.202512567)
Supplement: Supplementary file 1 — Supporting Information [file ADVS-12-e12567-s001.docx]

**Supporting Information**

**Dendrobium Officinale-Derived Carbon Dots Nanozymes Alleviate Colitis by Orchestrating Intestinal Mucus-Epithelium-Immune Barriers**

*Chenxi Xu^#^, Xiaoling Huang^#^, Zhichao Deng, Ruiying Wang, Seyedalireza Ghazimirsaeid Li Yao, Yuanyuan Zhu, Bowen Gao, Junlong Fu, Mingxin Zhang^*^, Mei Yang^*^, and Mingzhen Zhang**^*^*

M. Zhang

Department of Gastroenterology

The First Affiliated Hospital of Xi’an Medical University

Xi’an, Shaanxi, 710077, China

E-mail: [zmx3115@xiyi.edu.cn](mailto:zmx3115@xiyi.edu.cn)

M. Yang

Department of Organ Procurement and Allocation

The First Affiliated Hospital of Xi’an Jiaotong University

Xi’an, Shaanxi, 710061, China

E-mail: [meiyang@xjtu.edu.cn](mailto:meiyang@xjtu.edu.cn)

C. Xu, M. Zhang

Department of Hepatobiliary Surgery

the First Affiliated Hospital of Xi’an Jiaotong University

Xi’an, Shaanxi, 710061, China

E-mail: [mzhang21@xjtu.edu.cn](mailto:mzhang21@xjtu.edu.cn)

C. Xu, Z. Deng, R. Wang, S. Ghazimirsaeid, L. Yao, Y. Zhu, B. Gao, J. Fu, M. Zhang

School of Basic Medical Sciences

Xi’an Jiaotong University

Xi’an, Shaanxi, 710061, China

X. Huang

Department of Gastroenterology

People's Hospital of Xinjiang Uygur Autonomous Region

Urumqi, Xinjiang Uygur Autonomous Region, 830001, China

L. Yao

Department of Neurology

XD Group Hospital

Xi’an, Shaanxi, 710077, China

**Experimental Section**

*Evaluation of antioxidant capacity in DO-CD:* ⋅ABTS+ assay: Firstly, the ABTS solution was added to an oxidizing agent and allowed to react thoroughly overnight to generate an ABTS masterbatch. Then, the ABTS masterbatch was diluted 50-fold with PBS solution to form the ABTS working solution. DO-CDs at suitable concentrations (2.5, 5, 10, 15, and 40 μg/mL) were combined with ⋅ABTS⁺ radicals for minutes, and the absorbance at 734 nm was detected.

NBT assay: DO-CDs at varying concentrations (5, 10, 20, 40, and 80 μg/mL) were individually introduced into a solution containing riboflavin at a concentration of 20 μM, nitro blue tetrazolium (NBT) at 0.05 mM, and L - methionine (L - met) at 13 mM. After subjecting the mixtures to LED irradiation for 10 minutes, the absorbance at 560 nm was measured to evaluate the potential interactions.

TMB assay: Hydroxyl radicals (⋅OH) were generated by mixing the Fenton reagent, which comprised 10 μM Fe²⁺ and 50 μM H₂O₂. These ⋅OH radicals possess the oxidizing capability to convert 3,3',5,5'-tetramethylbenzidine (TMB) into its oxidized form, oxTMB. Subsequently, the generated ⋅OH radicals were incubated with TMB at a concentration of 0.3 mM and DO-CDs at various concentrations (10, 20, 40, 60, and 80 μg/mL). The absorbance of the reaction mixture at 645 nm was measured after 12 minutes to assess the extent of the oxidation reaction.

⋅DPPH radical assay: A solution of ⋅DPPH at a concentration of 125 μM in ethanol was separately combined with DO-CDs at various concentrations (1, 5, 10, 20, and 40 μg/mL). Following an incubation period of 30 minutes, the absorption spectra at 517 nm of the resulting mixtures were measured.

ESR assay: i) ⋅O₂⁻ radicals were produced by combining DTPA (1 mM), XOD (2.45 mM), and xanthine (0.4 mM) in PBS. Subsequently, BMPO (5 mg/mL) was introduced as a radical-trapping agent. Following the addition of DO-CDs and incubation at 37 °C for 10 minutes, electron spin resonance (ESR) spectra of the reaction mixtures were recorded. ii) For the trapping of ⋅OH radicals, a mixture of 5,5 - dimethyl - 1 - pyrroline - N - oxide (DMPO, 100 mM), FeSO₄ (50 μM), and H₂O₂ (10 μM) was prepared. Following the addition of DO-CDs at different concentrations and a reaction time of 3 minutes, the corresponding ESR spectra were obtained to characterize the formation and interaction of ⋅OH radicals with the added DO-CDs.

*Cell culture:* RAW 264.7 cells were maintained in a DMEM medium containing 10% fetal bovine serum (FBS), 100 U/mL penicillin, and streptomycin at 37°C under a 5% CO₂ atmosphere.


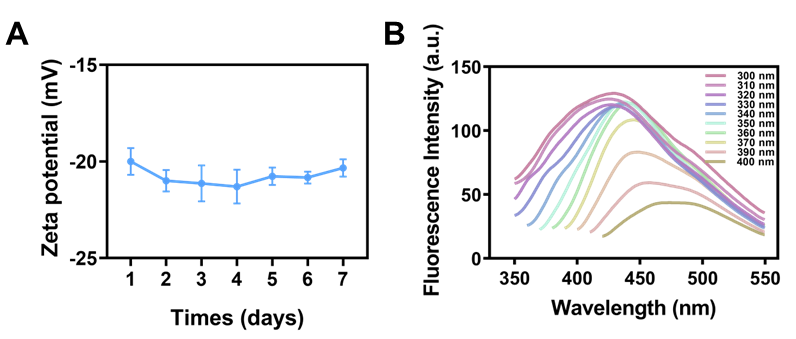


**Figure S1.** Characterization of DO-CDs. (A) The zeta-potential of DO-CDs was measured over seven consecutive days. (n = 3). (B) Fluorescence emission spectra of DO-CDs at different excitation wavelengths.


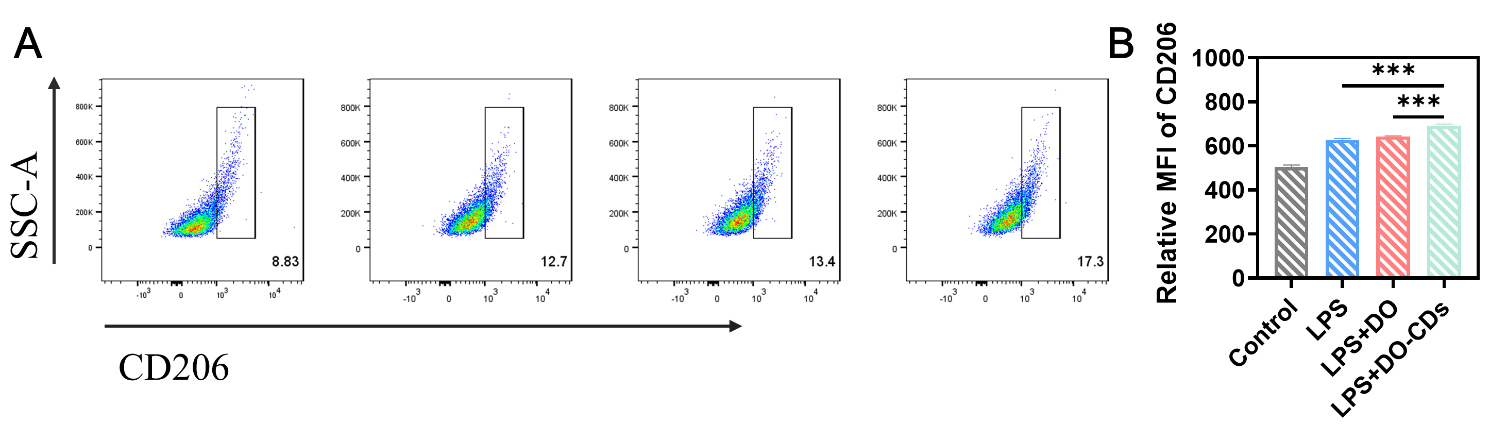


**Figure S2.** Flow cytometric analysis of M2-phenotype macrophages (A) and CD206 labeling quantification (B). (n = 3). Statistical significance was indicated as *p < 0.05, **p < 0.01, ***p < 0.001.


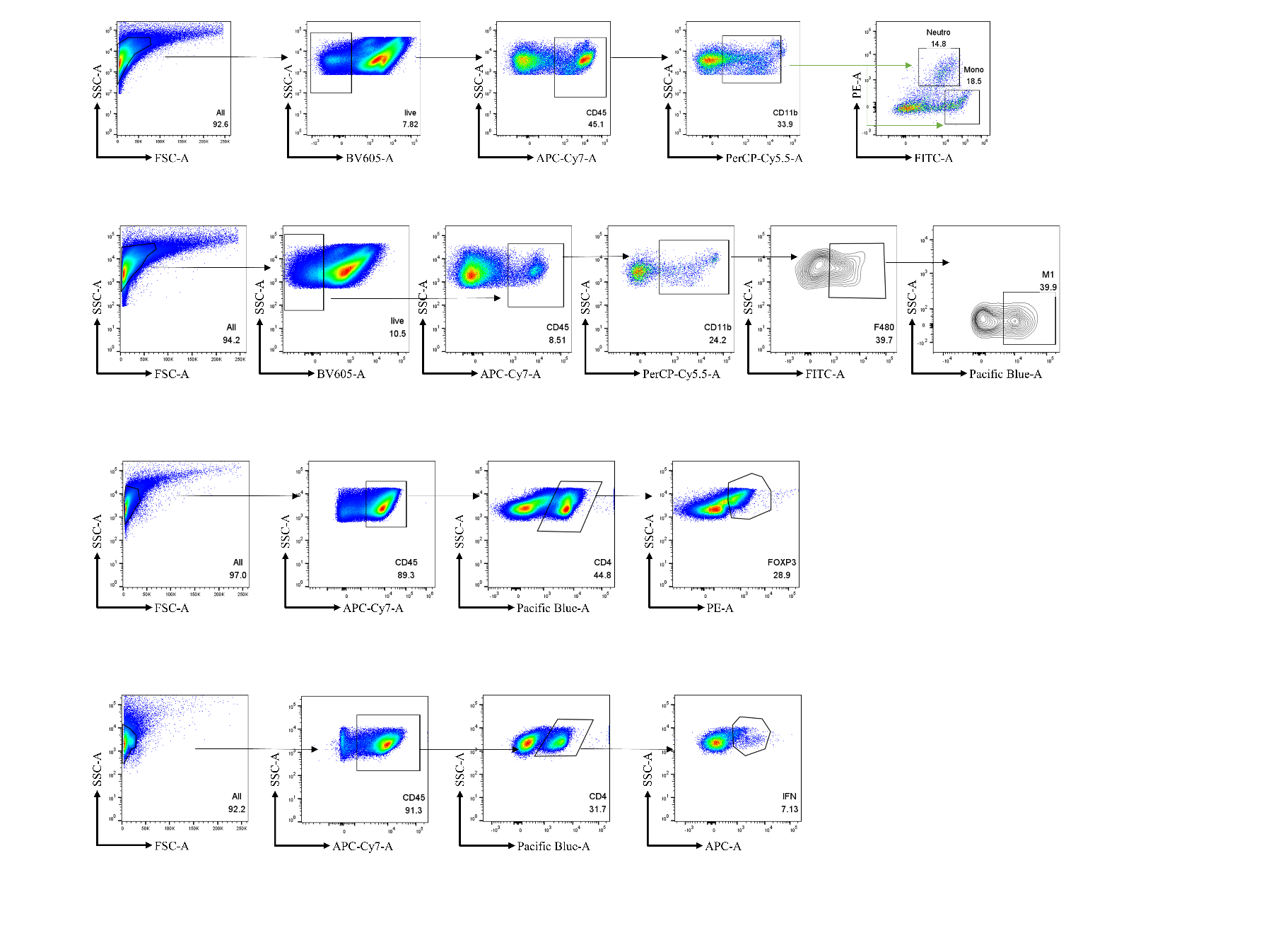


**Figure S3.** The gating strategy for flow cytometric analysis of neutrophils and Monocytes.


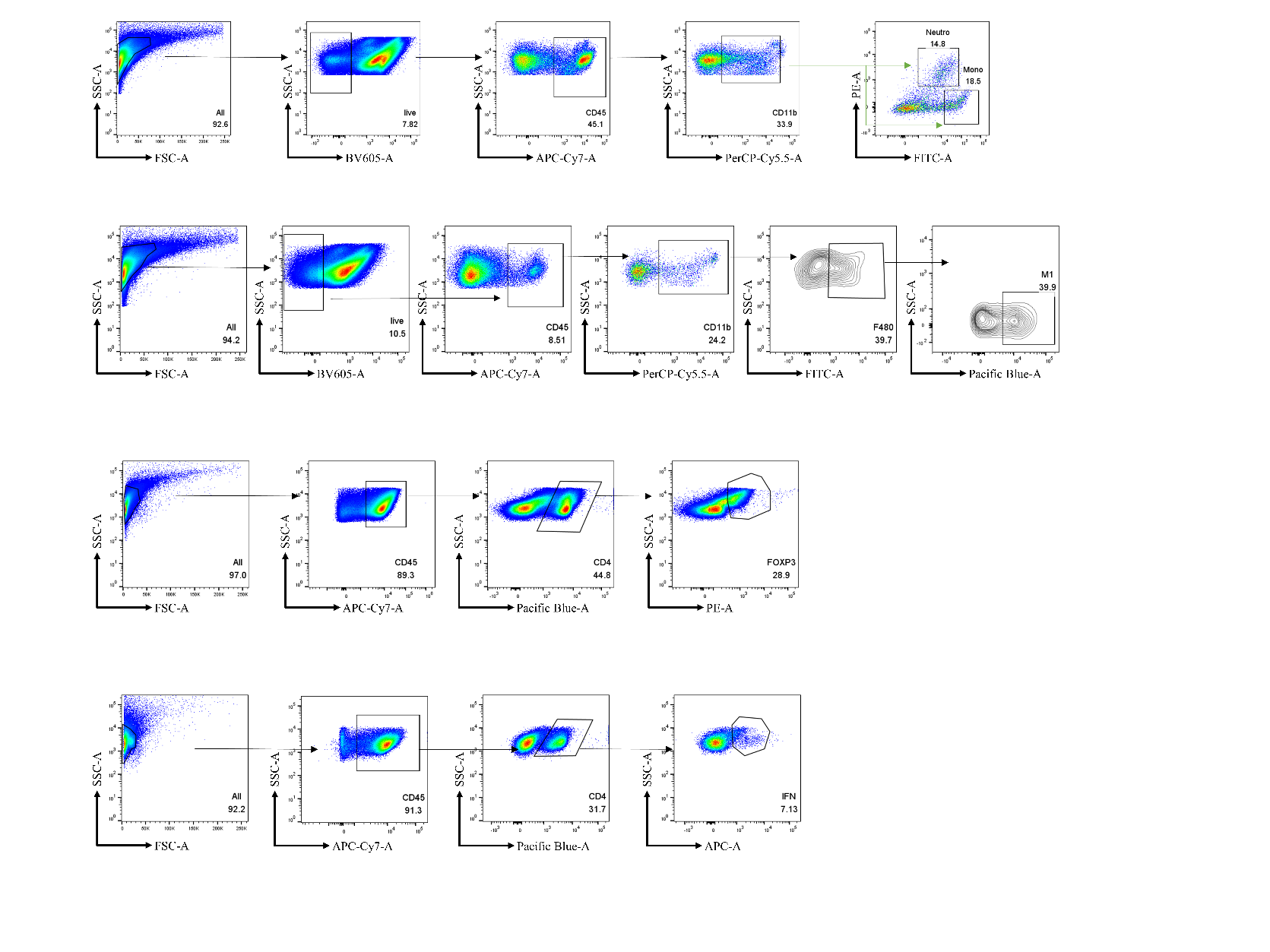


**Figure S4.** The gating strategy for flow cytometric analysis of M1-macrophages.


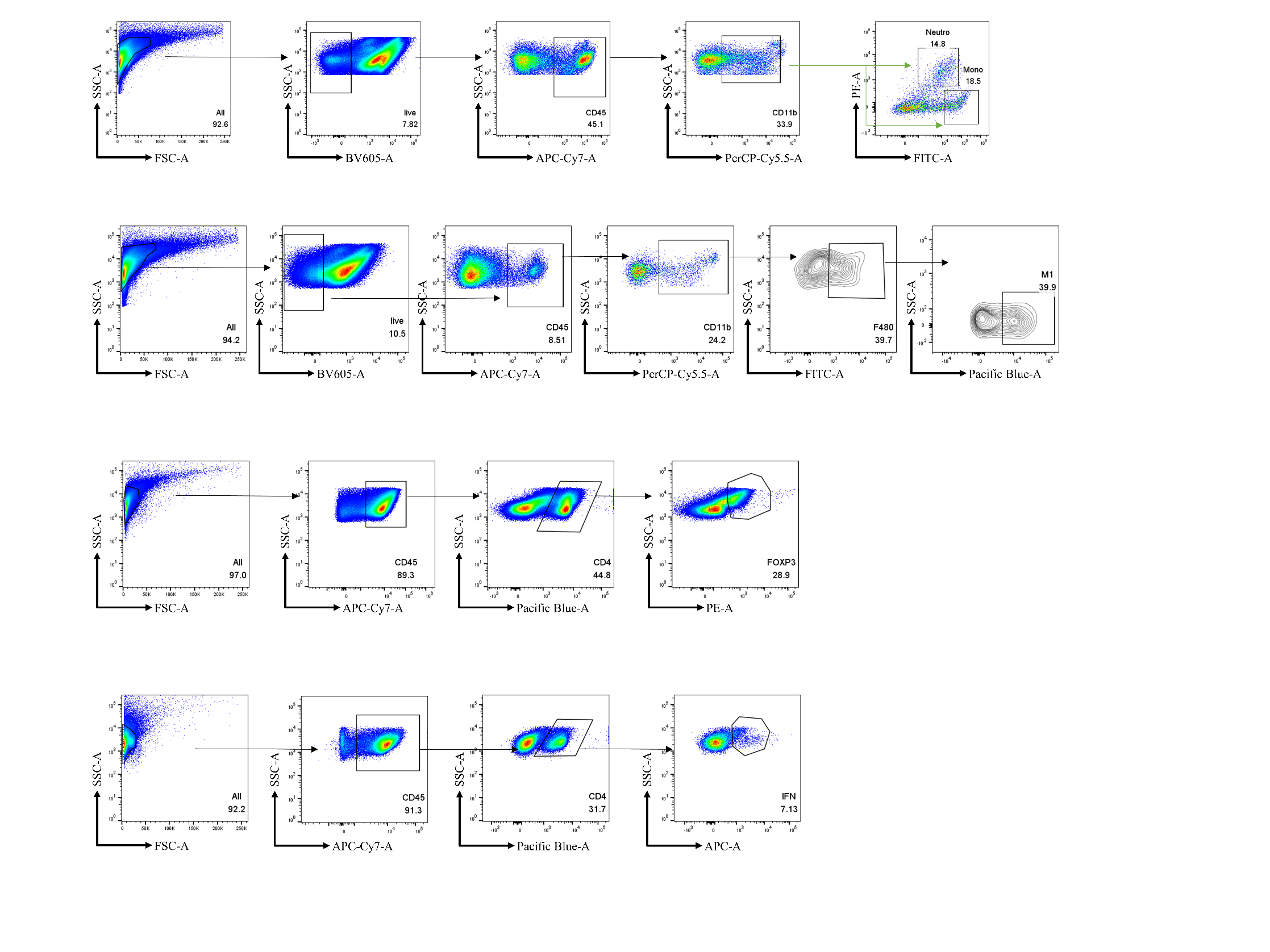


**Figure S5.** The gating strategy for flow cytometric analysis of Foxp3+ Treg cells.


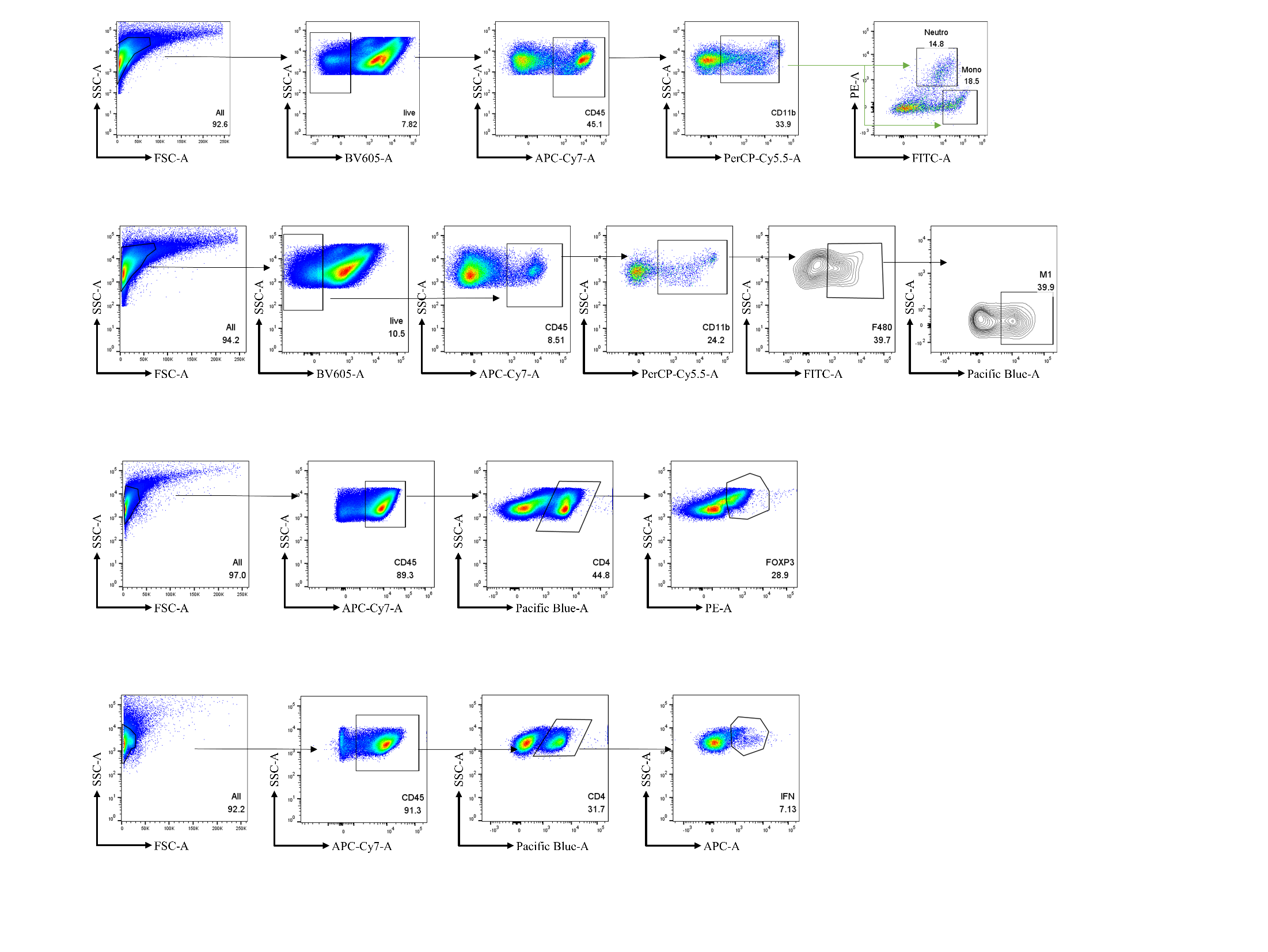


**Figure S6.** The gating strategy for flow cytometric analysis of IFN-γ+ Th1 cells.

**
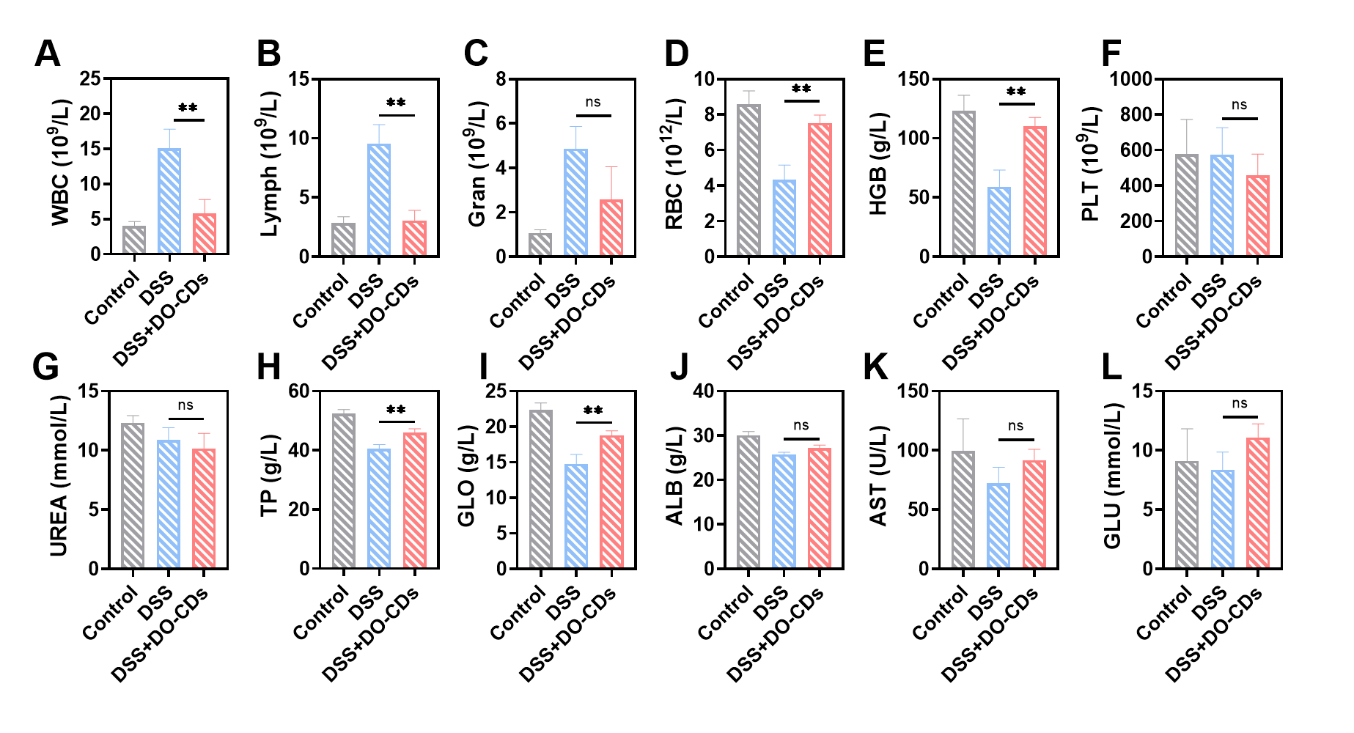
**

**Figure S7.** Blood biochemical and hematological parameters. (n = 3). Statistical significance was indicated as *p < 0.05, **p < 0.01, ***p < 0.001.

**Table S1.** Antibodies

| Number | Antibody | Clone | Brand |
| --- | --- | --- | --- |
| 1 | APC/Cyanine7 anti-mouse CD45 Antibody | 30-F11 | Biolegend |
| 2 | FITC anti-mouse Ly-6C | Hk1.4 | Biolegend |
| 3 | FITC anti-mouse F4/80 Antibody | BM8 | Biolegend |
| 4 | PE anti-mouse Ly-6G Antibody | 1A8 | Biolegend |
| 5 | PE anti-mouse CD206 (MMR) Antibody | C068C2 | Biolegend |
| 6 | PE anti-mouse FOXP3 Antibody | MF-14 | Biolegend |
| 9 | APC anti-mouse IFN-γ Antibody | XMG1.2 | Biolegend |
| 10 | APC anti-mouse CD11c Antibody | N418 | Biolegend |
| 11 | Pacific Blue™ antibody I-A/I-E Antibody | M5/114.15.2 | Biolegend |
| 12 | Pacific Blue™ anti-mouse CD4 Antibody | RM4-4 | Biolegend |
| 13 | PerCP/Cyanine5.5 anti-mouse/human CD11b Antibody | M1/70 | Biolegend |
